# Deleterious Rare Variants Reveal Risk for Loss of GABA_A_ Receptor Function in Patients with Genetic Epilepsy and in the General Population

**DOI:** 10.1371/journal.pone.0162883

**Published:** 2016-09-13

**Authors:** Ciria C. Hernandez, Tara L. Klassen, Laurel G. Jackson, Katharine Gurba, Ningning Hu, Jeffrey L. Noebels, Robert L. Macdonald

**Affiliations:** 1 Department of Neurology, Vanderbilt University, Nashville, Tennessee, 37232, United States of America; 2 Faculty of Pharmaceutical Sciences, University of British Columbia, Vancouver, British Columbia, V6S 1Z3, Canada; 3 Program in Neuroscience, Vanderbilt University, Nashville, Tennessee, 37232, United States of America; 4 Department of Neurology, Baylor College of Medicine, Houston, Texas, 77030, United States of America; Odense University Hospital, DENMARK

## Abstract

Genetic epilepsies (GEs) account for approximately 50% of all seizure disorders, and familial forms include mutations in single GABA_A_ receptor subunit genes (*GABRs)*. In 144 sporadic GE cases (GECs), exome sequencing of 237 ion channel genes identified 520 *GABR* variants. Among these variants, 33 rare variants in 11 *GABR* genes were present in 24 GECs. To assess functional risk of variants in GECs, we selected 8 variants found in *GABRA*, 3 in *GABRB*, and 3 in *GABRG* and compared them to 18 variants found in the general population for *GABRA1* (n = 9), *GABRB3* (n = 7), and *GABRG2* (n = 2). To identify deleterious variants and gain insight into structure-function relationships, we studied the gating properties, surface expression and structural perturbations of the 32 variants. Significant reduction of GABA_A_ receptor function was strongly associated with variants scored as deleterious and mapped within the N-terminal and transmembrane domains. In addition, 12 out of 17 variants mapped along the β+/α- GABA binding interface, were associated with reduction in channel gating and were predicted to cause structural rearrangements of the receptor by *in silico* simulations. Missense or nonsense mutations of *GABRA1*, *GABRB3* and *GABRG2* primarily impair subunit biogenesis. In contrast, *GABR* variants affected receptor function by impairing gating, suggesting that different mechanisms are operating in *GABR* epilepsy susceptibility variants and disease-causing mutations. The functional impact of single *GABR* variants found in individuals with sporadic GEs warrants the use of molecular diagnosis and will ultimately improve the treatment of genetic epilepsies by using a personalized approach.

## Introduction

GABA_A_ receptors are hetero-pentameric, ligand-gated chloride channels that mediate both phasic inhibitory synaptic transmission and tonic perisynaptic inhibition in the brain. They assemble from combinations of nineteen subunit subtypes (α1–6, β1–3, γ1–3, δ, ε, θ, π and ρ1–3) that influence channel properties and timing of GABA_A_ receptor-mediated inhibitory post-synaptic currents [1]. The 1000 human genome project identified 7,011 variants in coding regions of α1, α4–6, β1–3, γ1–3 GABA_A_ receptor subunits (The 1000 Genomes Project) [2]. However, only 24 non-synonymous GABA_A_ receptor subunit variants are disease-causative in monogenic cases of genetic epilepsy (GE), whereas 3 were found in non-monogenic cases [3]. Genetic epilepsies (GEs) account for approximately 50% of all epilepsies diagnosed worldwide [4], and familial forms include mutations in single GABA_A_ receptor subunit genes (*GABRs)*. Monogenic cases of GEs are associated primarily with mutations in a subset of *GABRs*, *GABRA1*, *GABRB3*, *GABRG2*, which compose one of the predominant receptor isoforms *in vivo* (α1β3γ2) [5, 6]. Disruption of key assembly, trafficking or gating motifs results in overall reduction of α1β3γ2 GABA_A_ receptor currents in *in vitro* studies [3, 7–12].

Sporadic GEs with predicted complex gene variant profiles are more difficult to interpret due to the still poorly understood contribution of deleterious GABA_A_ receptor gene variants present in both affected and unaffected individuals. Complex inheritance in GEs implies that a combination of multiple susceptibility alleles and environmental factors contribute to the penetrance and expressivity in affected individuals, which are assumed to be normally distributed in the population, and none of which has sufficient effect to segregate through large families when acting by itself [13]. Although complex polygenic inheritance is likely associated with most genetic epilepsy syndromes, rare monogenic mutations of transmembrane ion channels associated with GEs in several large pedigrees and in sporadic cases with de novo mutations have also been identified [14–17]. To date, three studies have reported the presence of mutations in other *GABRs* associated with epilepsy. An inherited mutation in the α6 subunit, R46W, was identified in a patient with childhood absence epilepsy and atonic seizures from a generalized epilepsy with febrile seizures plus (GEFS+) cohort [17] and reported to cause impairment in both channel gating and cell surface expression [18]. The *de novo* mutation β1(F246S) was identified in a case of infantile spasms [6], whereas a *de novo* mutation in exon 4 of *GABRB2* (β2(M79T)) was discovered in a patient with intellectual disability and epilepsy [19]. These findings suggest that additional *GABRs* may be “epilepsy” genes and that other *GABRs* may also contribute to the final clinical presentation as polygenic and/or modifying genes of lesser effect.

Given the prevalence of *GABR* variants in individuals with and without epilepsy, we studied the gating properties, surface expression and structural perturbations of 14 rare variants identified in *GABRs* and found in individuals with GE in the exome sequencing of 237 ion channel genes project [20] and 18 rare variants found in individuals from the general population in *GABRA1*, *GABRB3* and *GABRG2* in the NHLBI Exome Sequencing Project (ESP variants). We employed whole cell patch clamp recordings and flow cytometry from HEK293T cells transfected with combinations of wild type and mutant GABA_A_ receptor subunits to quantify changes in macroscopic GABA-evoked currents and surface and total expression levels and 3-D homology modelling to determine structure function relationships. We found that variants that were mapped to the β+/α- subunit-subunit interface within the N-terminal and transmembrane domains of the GABA_A_ receptor were more deleterious than those located in the γ+/β-, α+/β-, or α+/γ- subunit-subunit interfaces. Indeed, these variants substantially decreased current amplitude, an effect not caused by reduced surface expression of functional receptors. They did, however, slow activation substantially and accelerate channel deactivation, suggesting an impairment in gating. These findings were confirmed by structural simulations comparing wild type and mutant receptors, showing that these variants caused significant structural perturbations through canonical motifs that couple ligand binding to channel gating.

Since GABA_A_ receptors are critical to excitability regulation, the presence of deleterious loss-of-function *GABR* variants can lead to neuronal disinhibition, promote hyperexcitability, and lead to GE. These variants likely produce disinhibition primarily by gating impairment mechanisms. In this work, we propose that functional defects in one or multiple *GABRs* might confer additive risk for sporadic GEs and help predict the risk of loss of GABAergic function in epileptic and non-epileptic subjects.

## Methods and Materials

### Study design

The exome sequencing of 237 ion channel genes project [20] identified 520 non-synonymous variants in *GABRs* in 144 GE cases (GECs) and 147 non-synonymous variants in 42 healthy individuals (Table 1). Among 667 variants identified (520 in GECs and 147 in healthy controls), 35 variants representing 11 different *GABRs* were unique (33 unique variants in GECs (Table 2) and 2 unique variants in healthy controls), and were found to be correlated with the 24 GEC variants (p *=* 0.0120, Fisher’s exact test). To assess the functional risk of rare variants in GECs, we selected 14 variants found in *GABRA1* (*n =* 1), *GABRA4* (*n =* 2), *GABRA5* (*n =* 4), *GABRA6* (*n =* 1), *GABRB1* (*n =* 1), *GABRB2* (*n =* 2), *GABRG1* (*n =* 2) and *GABRG3* (*n =* 1) (Table 3, see Results section). We excluded GABA_A_ receptor subunit variants derived from 3 *GABRs* (*GABRE*, *GABRP*, *GABRR2*), whose distribution throughout the brain and receptor stoichiometry are uncertain [21]. For assessment of the general population, we selected an additional 18 variants found in this group of individuals (ESP variants) (NHLBI Exome Sequencing Project, Seattle, WA. URL: http://evs.gs.washington.edu/EVS/ release May-14 2015) within the monogenic epilepsy associated *GABRA1* (*n =* 9), *GABRB3* (*n =* 7) and *GABRG2* (*n =* 2) with frequency <0.5% [2]. The selected GEC and ESP variants were located in protein-coding regions of *GABRs* that were within four defined GABA_A_ receptor structural regions: signal peptide, N-terminal domain, M3/M4 cytoplasmic loop, and transmembrane domain. Finally, the impact of the *GABR* variants on protein structure were scored with PolyPhen-2 [22], SIFT [23] and SignalP-V2.0 [24]. Only PolyPhen-2 scores were reported (Table 3, see Results section).

**Table 1:** Comparison of total number of *GABR* variants in controls and GECs in the 237 ion channel genes project^1^.

Number of individuals in the 237 ion channel genes project^1^	Total number of *GABR* variants	Number of unique^2^ variants by *GABR* gene	Occurrence of unique^2^ variants
42 controls	147	2	2
144 GECs	520	24^3^	33^4^

^1^GECs = genetic epilepsy cases [20].

^2^Unique = *GABR* variants that were mutually exclusive among controls and GECs.

^3^See Table 2, column 1 for details.

^4^See Table 2, column 3 for details.

**Table 2:** Unique *GABR* variants from GECs reported in the 237 ion channel genes project^1^.

*GABR* gene	Variant	Occurrence of variants among GECs
*GABRA1*	T20I*	1
*GABRA4*	H372P*	1
*GABRA5*	W280R*	3
*GABRA5*	P453L*	1
*GABRB2*	R293W*	1
*GABRG3*	A303T*	1
*GABRA4*	A19T*	1
*GABRA5*	V204I*	1
*GABRA5*	S402A*	1
*GABRA6*	Q237R*	1
*GABRB1*	H421Q*	1
*GABRB2*	R354C*	2
*GABRG1*	S16R*	1
*GABRG1*	S414N*	1
*GABRE*	R472H	1
*GABRE*	S484L	1
*GABRP*	R200H	2
*GABRP*	S292P	1
*GABRP*	S293P	1
*GABRP*	R389N	1
*GABRR2*	R287H	1
*GABRR2*	V294I	2
*GABRE*	R452G	1
*GABRP*	V349A	5

^1^GECs = genetic epilepsy cases [20].

*GABR variants characterized in this study.

**Table 3:** Predicted and observed functional effects of missense variants in *GABR* genes in patients with genetic epilepsy and in the general population. GECs = genetic epilepsy cases. ESP = Exome Sequencing Project. SP = signal peptide. CL = M3/M4 cytoplasmic loop. TM = transmembrane. NT = N-terminal.

*GABR* gene	Variant	Phenotype category	PolyPhen-2 category	HumDivscore^1^	HumVarscore^1^	Domain position	Reducedcurrent^2^	Gatingdefect^3^
*GABRA1*	T20I	GECs	benign	0.001	0.003	SP	no	yes
*GABRA4*	H372P	GECs	benign	0.0	0.0	CL	yes	no
***GABRA5***	**W280R**	**GECs**	**damaging**	**1.0**	**0.999**	TM	yes	yes
*GABRA5*	P453L	GECs	possibly	0.515	0.117	TM	no	yes
***GABRB2***	**R293W**	**GECs**	**damaging**	**1.0**	**1.0**	TM	yes	yes
***GABRG3***	**A303T**	**GECs**	**damaging**	**0.999**	**0.966**	TM	yes	yes
*GABRA4*	A19T	GECs	benign	0.001	0.001	SP	no	no
*GABRA5*	V204I	GECs	benign	0.001	0.005	NT	yes	yes
*GABRA5*	S402A	GECs	benign	0.0	0.004	CL	no	no
*GABRA6*	Q237R	GECs	benign	0.041	0.06	NT	yes	yes
*GABRB1*	H421Q	GECs	benign	0.002	0.006	CL	no	no
***GABRB2***	**R354C**	**GECs**	**damaging**	**0.999**	**0.892**	CL	yes	yes
*GABRG1*	S16R	GECs	benign	0.023	0.029	SP	no	no
*GABRG1*	S414N	GECs	possibly	0.57	0.334	CL	no	no
*GABRA1*	D9E	ESP	benign	0.0	0.0	SP	no	no
*GABRA1*	P29S	ESP	benign	0.02	0.01	NT	yes	no
***GABRA1***	**H129Y**	**ESP**	**damaging**	**0.976**	**0.880**	NT	yes	no
***GABRA1***	**R147W**	**ESP**	**damaging**	**1.0**	**1.0**	NT	no	yes
*GABRA1*	T371I	ESP	benign	0.237	0.119	CL	no	no
*GABRA1*	D383N	ESP	benign	0.165	0.024	CL	no	no
*GABRA1*	P409S	ESP	benign	0.005	0.011	CL	yes	no
*GABRA1*	K410R	ESP	benign	0.121	0.069	CL	no	no
***GABRA1***	**T441M**	**ESP**	**damaging**	**1.0**	**0.999**	TM	yes	yes
*GABRB3*	R194Q	ESP	benign	0.016	0.009	NT	yes	yes
*GABRB3*	D197N	ESP	benign	0.001	0.006	NT	no	yes
***GABRB3***	**V200I**	**ESP**	**damaging**	**0.945**	**0.646**	NT	yes	no
*GABRB3*	R221K	ESP	benign	0.0	0.002	NT	yes	yes
*GABRB3*	R238W	ESP	benign	0.161	0.033	NT	yes	yes
*GABRB3*	D387N	ESP	benign	0.03	0.027	CL	yes	yes
***GABRB3***	**I448V**	**ESP**	**damaging**	**0.718**	**0.447**	TM	yes	no
***GABRG2***	**L57F**	**ESP**	**damaging**	**0.995**	**0.962**	NT	yes	no
*GABRG2*	A402T	ESP	possibly	0.534	0.205	CL	no	no

^1^PolyPhen-2 scores are shown for HumDiv model that evaluates rare alleles, and HumVar model for distinction of variants with drastic effects from all the remaining human variation. The variants defined as deleterious are highlighted in bold.

^2,3^Refers to Tables 4 and 5.

### cDNA constructs, cell culture and transfections

cDNAs encoding human α1, α4, α5, α6, β1, β2, β3, γ1, γ2 and γ3 GABA_A_ receptor subunit subtypes (GenBank accessions NM000806, NM000809, NM000810, NM000811, NM000812, NM000813, NM021912, NM173536, NM198904, and NM033223, respectively) were subcloned into the plasmid expression vector pcDNA3.1 (Thermo Fisher Scientific, Waltham, MA) using standard techniques. *GABR* variants were generated by site-directed mutagenesis using the QuikChange Site-Directed Mutagenesis Kit (Agilent Technologies, Santa Clara, CA) and verified by sequencing. FLAG (DYKDDDDK) or HA (YPYDVPDYA) epitopes were inserted between amino acids 4 and 5 of the mature GABA_A_ receptor subunit subtypes as needed, so that subunit total and cell surface expression could be determined by flow cytometry.

Human embryonic kidney cells (HEK293T) were grown in 100 mm tissue culture dishes (Corning. Corning, NY) with DMEM, supplemented with 10% fetal bovine serum at 37C in 5% CO2 / 95% air and passaged every 3–4 d. Wild type and variant (var) subunits were co-expressed in HEK293T cells with the following GABA_A_ receptor subunit combinations: wild type α1β3γ2, α1β2γ2, α4β2γ2, α5β3γ2, α6β2γ2, α1β1γ2, α5β3γ1, and α5β3γ3; and variant α1(var)β3γ2, α1β3(var)γ2, α1β3γ2(var), α1(var)β2γ2, α4(var)β2γ2, α5(var)β3γ2, α6(var)β2γ2, α1β1(var)γ2, α1β2(var)γ2, α5β3γ1(var), and α5β3γ3(var). For electrophysiological experiments, cells were plated onto non-coated cover glass chips and transfected with 0.3 μg of each α, β, and γ subunit (wildtype or *GABR* variants), and 0.05 μg cDNA of EGFP (to identify transfected cells) using the FuGENE 6 transfection reagent (Promega, Madison, WI, 3 μl per μg cDNA) according to the manufacturer’s instructions. For surface expression measurement using flow cytometry, 4 x 10^5^ cells were plated onto 60-mm diameter culture dishes. Twenty-four hours after plating, cells were transfected with EGFP and GABA_A_ receptor subunit cDNAs using 3.0 μg of polyethylenimine (PEI, MW 40,000, Polysciences, Warrington, PA) per 1 μg of cDNA. Wild type and experimental conditions included 0.1 μg of EGFP cDNA and 1 μg of each α, β, and γ subunit cDNA. Experiments were performed over the subsequent 2–3 days.

### Whole cell electrophysiology

Whole cell recordings from lifted HEK293T cells were obtained at room temperature and the external solution was composed of (in mM): 142 NaCl, 8 KCl, 10 D(+)-glucose, 10 HEPES, 6 MgCl_2_.6H_2_O, and 1 CaCl_2_ (pH 7.4, ~326 mOsm). The internal solution consisted of (in mM): 153 KCl, 10 HEPES, 5 EGTA 2 Mg-ATP, and 1 MgCl_2_.6H_2_O (pH 7.3, ~300 mOsm). The Cl^-^ reversal potential was near 0 mV, and cells were voltage clamped at -20 mV. 1 mM GABA was applied using a four-barrel square glass pipette connected to a SF-77B Perfusion Fast-Step system (Warner Instruments Corporations). The solution exchange time across the open electrode tip was ∼200–400 μs, and the exchange around lifted cells (~8–10 pF) occurred within 800 μs, which was sufficiently fast for these experiments [25] and guaranteed rapid solution exchanges and accurate measure of the kinetic properties of the receptor. All experiments were performed at room temperature (22–23°C). Whole cell currents were amplified and low-pass filtered at 2 kHz using an Axopatch 200B amplifier, digitized at 10 kHz using Digidata 1550, and saved using pCLAMP 10.4 (Axon Instruments). Data were analysed offline using Clampfit 10.4 (Axon Instruments). Activation onset and deactivation weight time constants (τ) were measured from currents obtained by application of 1 mM GABA for 10 ms, while peak current amplitude was measured from currents obtained by application of 1 mM GABA for 4 s. Activation and deactivation time constants (τ) were fitted using the Levenberg–Marquardt least squares method with up to four component exponential functions of the form ∑*a*_*n*_exp(–*t*/τ_*n*_) +*C*, where *n* is the number of the exponential components, *t* is time, *a* is the relative amplitude, τ_*n*_ is the time constant, and *C* is the residual current at the end of GABA application. Additional components were accepted only if they significantly improved the fit, as determined by an *F* test on the sum of squared residuals. The multiexponential time course of deactivation was presented as a weighted time constant, defined by the following expression: ∑*a*_*n*_τ_*n*_/∑*a*_*n*_. Observable changes in current time constants (τ) caused by variations in activation onset and deactivation weigh time constants (τ) caused by variants were computed by measuring the gating impairment ratio, which was computed as follow: Activation τ_variant_ / τ_wild type_ / Deactivation τ_variant_ / τ_wild type_. GABA_A_ receptor current concentration–response curves were fitted using GraphPad Prism version 6.07 for Windows (GraphPad Software, La Jolla, CA). Inhibition of 1 mM GABA_A_ receptor evoked currents by 10 μM zinc was measured by pre-application for 10 s followed by co-application with GABA for 4 s. GABA and zinc were obtained from Sigma.

### Flow cytometry

Cells were harvested ~48 hours after transfection using 37°C trypsin-EDTA and placed immediately on ice in FACS buffer (Ca^2+^/Mg^2+^ -free PBS with 2% FBS and 0.05% NaN_3_) and transferred to 96-well polystyrene V-bottom plates. GABA_A_ receptor subunits were detected with antibodies to human α1 (N-terminal clone BD24, EMD Millipore, Billerica, MA), human β2/3 (N-terminal clone BD17, EMD Millipore), the HA epitope tag (clone 16B12, Covance Laboratories, Nashville, TN), and the FLAG epitope tag (F7425, Sigma-Aldrich, St. Louis, MO). Following primary antibody incubation, cells were washed four times with FACS buffer and incubated with anti-mouse or anti-rabbit IgG1 secondary antibody conjugated to the Alexa647 fluorophore (Life Technologies, Carlsbad, CA) before additional washing and fixation with 2% w/v paraformaldehyde, 1mΜ EDTA diluted in PBS. Total cellular protein detection began with permeabilization by 15 min incubation with Cytofix/Cytoperm (BD Biosciences, San Jose, CA). Cells were washed twice with 1x PermWash (BD Biosciences) before primary antibody incubation. All antibodies were diluted in PermWash. After secondary antibody incubation, cells were washed five times with PermWash and twice in FACS buffer before fixation. Fluorescence intensity (FI) of all samples was determined using an LSR II 3-laser flow cytometer (BD Biosciences) and analysed with FlowJo 7.6 (Flowjo, Ashland, OR). EGFP expression was used as an indicator of successful transfection; therefore, the primary gate selected the EGFP-positive population of cells. Subsequent gates were applied to exclude debris and doublets. For all experiments, the net FI of samples was determined by subtracting the mean FI of cells transfected with empty vector from the mean FI of cells expressing GABA_A_ receptor subunits. The relative FI for each condition was calculated by normalizing the net FI of each experimental condition to the net FI of cells expressing wild type subunits.

### Structural modelling and simulations

GABA_A_ receptor subunits sequences were loaded into Swiss-PdbViewer 4.10 [26] for template searching against the ExPDB database (ExPASy, http://www.expasy.org/). The structure of the *Caenorhabditis elegans* glutamate-gated chloride channel gene (GluCl; PDB: 3RHW) [27] was identified as the best template resulting in 33%, 36% and 41% sequence identity for *GABRG2*, *GABRA1* and *GABRB3*, respectively (similar results were obtained for other *GABRs*). The initial sequence alignments between GABA_A_ receptor subunits and *C*. *elegans* GluCl subunits were generated with full-length multiple alignments using ClustalW. Sequence alignments were inspected manually to assure accuracy among structural domains solved from the template. Because the long M3/M4 cytoplasmic loop of the GABA_A_ receptor subunits was absent in the solved GluCl structure, the corresponding fourth transmembrane domains (M4) were misaligned onto the template. Consequently, the former was excluded from the modelling, and separate alignments were generated for the M4 domains. Then full-length multiple alignments were submitted for automated comparative protein modelling implemented in a module incorporated in SWISS-MODEL (http://swissmodel.expasy.org/SWISS-MODEL.html). Before energy minimization, resulting structural models of human GABA_A_ receptor subunits were inspected manually, their structural alignments confirmed, and evaluated for proper h-bonds, presence of clashes and missing atoms using Molegro Molecular Viewer (CLC bio, Aarhus, Denmark). Then, pentameric GABA_A_ receptor models were generated by combining α, β and γ structural models in the stoichiometry 2β:2α:1γ with the subunit arrangement γ-β-α-β-α when viewed from the synaptic cleft. Neighbourhood structural conformational changes caused by a single mutated amino acid residue (variant) in GABA_A_ receptor subunits were simulated using Rosetta 3.1 [28], implemented as the Backrub module (https://kortemmelab.ucsf.edu). This method allowed the “repacking” of neighbouring residues within a radius of 6Å of the mutated residues. Up to twenty of the best-scoring structures were generated each time by choosing parameters recommended by the application. We measured mutation-induced structural differences by analysing the root mean squared (RMS) deviation between the initial (wild type) structures and superimposed simulated (mutated) structures. RMS deviation provided carbon-α/carbon-α, secondary structure, and side chain comparisons between two structurally aligned models, and the three parameters included the number of atoms altered; the larger the RMS deviation, the more the mutant structure deviated from the wild type structure. For each mutation, the average RMS deviation over the ten lowest energy structures was computed. Only RMS deviation of secondary structures and side chains were reported since the RMS deviations of carbon-α/carbon-α perturbations were similar between those reported for side chain perturbations. For *GABRB3* variants, RMS deviation was computed for structural models built based on both the GluCl (PDB: 3RHW)[27] and the human GABA_A_R-β3 (PDB: 4COF)[29] crystal structures (S1 Fig and S2 Fig). The structural alignment between the GABA_A_-β3 model and the human GABA_A_R-β3 crystal structure had a carbon α root mean square (CαRMS) of 1.29 Å for 292 atoms of the aligned amino acids. There were no major structural differences between the simulations of β3 subunit variants based on each respective model (S1 Fig and S2 Fig). The models were rendered using USF Chimera version 1.10 [30].

### Statistical Analysis

Numerical data were expressed as mean ± SEM. Statistical analysis was performed using GraphPad Prism (GraphPad Software 6.07). Statistical significance was taken as p < 0.05, using unpaired two-tailed Student's *t* test and one-way ANOVA with Dunnett’s multiple comparisons test as appropriate. Fisher’s Exact Test (two-tailed) was used to analyse statistical association between deleterious variants, structural domains and GABA_A_ receptor function.

## Results

### *GABR* variants were located within the structural domains of GABA_A_ receptors

**Fig 1:**
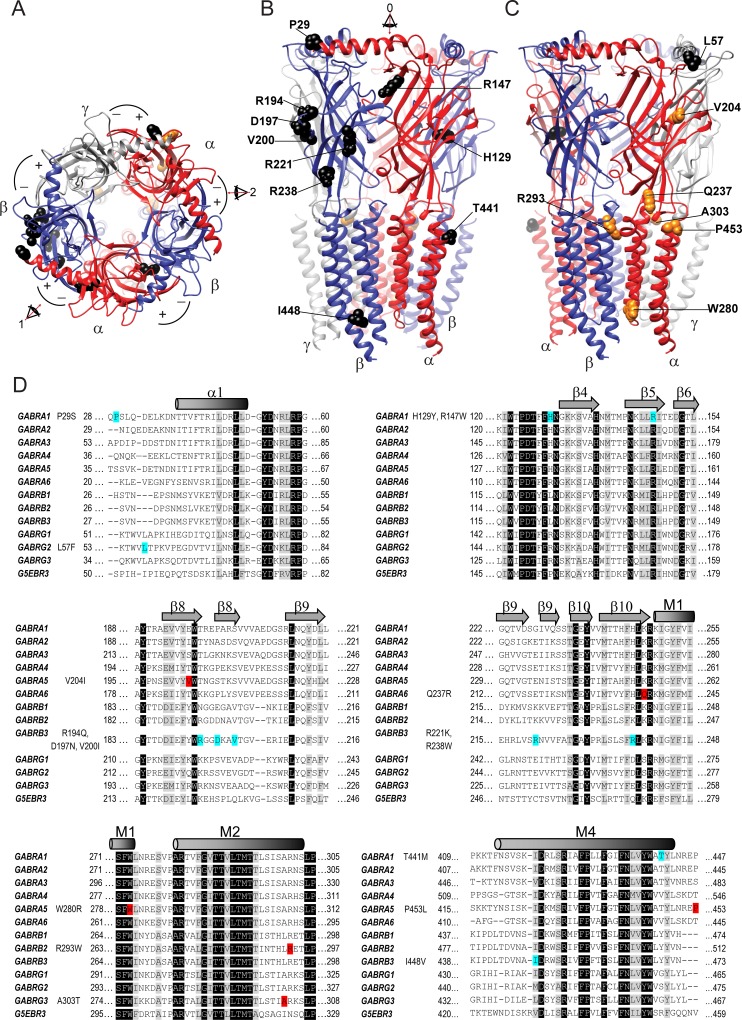
Mapping of *GABR* variants on the GABA_A_ receptor. (A), 3-D structural model of the GABA_A_ receptor rendered with subunits arranged in a γ-β-α-β-α counter clockwise sequence as viewed from the synaptic cleft. The principal (+) and complementary (-) interfaces of each subunit are shown with the β subunits in blue, α subunits in red and γ subunit in grey. Eye arrows 1 and 2 at the β+/α- interfaces match the view of the models in panels B and C, respectively. (B, C), show side views of the full-length pentameric receptor structure at the β+/α- subunit interfaces mapping GEC (orange) and ESP (black) *GABR* variants. Eye arrow *0* indicates the view of the 3-D model in panel (A). (D) Multiple sequence alignment of *GABR* genes and the solved GluCl crystal structure (*G5EBR3*) as a reference for structural domains of the GABA_A_ receptor. Locations of variants are highlighted in red for GEC and blue for ESP subunit variants. Secondary structures are indicated across *GABR* genes above the alignments, identical residues are highlighted in black and conserved residues in light grey. Each panel represents the succession in the sequence alignment where variants were found. Variants located in the signal peptide and M3/M4 cytoplasmic loop domains are not included.

To determine the location of *GABR* variants in the pentameric GABA_A_ receptor structure, we built 3-D homology models with subunit arrangement γ-β-α-β-α (Fig 1A–1C). We compared multiple sequence alignments among α, β, and γ GABA_A_ receptor subunits and the α-subunit of the glutamate-gated chloride channel (*G5EBR3*, GluCl; Fig 1D) [27]. GABA_A_ receptor subunits have a ∼200 residue N-terminal domain composed of a core of ten β-strands and four transmembrane α-helices (M1, M2, M3, M4) that are homologous to the GluCl receptor (Fig 1B and 1C). The N-terminal domain contains two ligand binding sites that are formed at the interface of two adjacent subunits between the principal (+) side of the β subunit and the complementary α subunit (−) side of the (i.e. β+/α- interface). Moreover, the β subunit complementary sides participate in the formation of the γ+/β- and α+/β- interfaces. The only interface without a β subunit is the α+/γ- interface which contains the binding site for benzodiazepines (Fig 1A).

Variants are located in the N-terminal (34%), transmembrane (19%), and M3/M4 cytoplasmic (34%) receptor domains (Fig 1 and Table 3) and signal peptide (13%). Most variants in the N-terminal domain were positioned in the outer β-strands (β4, β5, β8 and β9) and adjoining loops (Fig 1D). *In silico* analysis using Polyphen-2 [22] and SIFT [23], software programs that analyse mutation tolerance based on sequence conservation and local structural features, predicted that ~60% of variants located within the N-terminal and transmembrane domains would not be tolerated and potentially disrupt protein structure. Due to this negative effect on protein structure, these variants were classified as deleterious (Table 3). The variants located in the signal peptide, which is not present in the mature subunit, did not alter subunit function by *in silico* analysis using SignalP-V2.0 [24].

### Deleterious *GABR* variants reduced GABA-evoked currents with no reduction in receptor surface expression

To determine whether variants altered receptor function or biogenesis, we investigated GABA_A_ receptors containing each of the 32 variants identified (18 ESP and 14 GEC *GABR* variants) using whole cell patch clamp recordings and flow cytometry.

**Fig 2:**
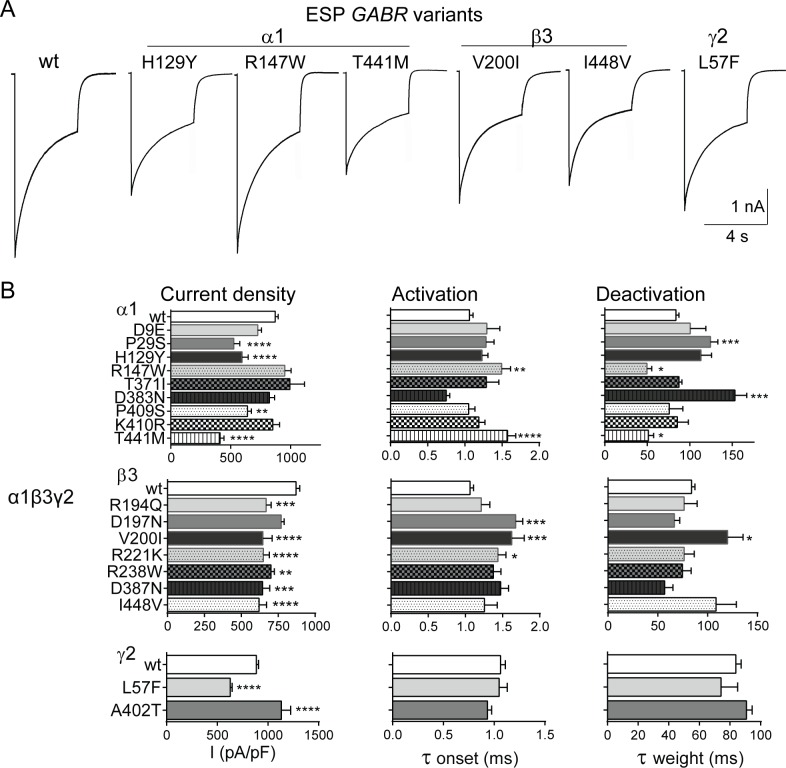
Deleterious ESP *GABR* variants caused major reductions of GABA-evoked currents. (A), Representative current traces from wild type (wt) and variant-containing receptors are presented. The variants α1H129Y, α1R147W, α1T441M, β3V200I, β3I448V, and γ2L57F were scored as deleterious by PolyPhen-2. GABA-evoked currents are the responses to 4 s pulses of 1 mM GABA on lifted cells expressing α1β3γ2 GABA_A_ receptors. (B), Bar plots summarize the effects of wt and ESP subunit variants on macroscopic parameters of GABA_A_ receptor currents. Current density was measured after 4 s pulses of 1 mM GABA, while activation and deactivation kinetics were measured after 10 ms pulses of 1 mM GABA. Values are expressed as mean ± S.E.M. *, **, *** and **** indicate p < 0.05, p < 0.01, p < 0.001 and p < 0.0001 respectively (one-way ANOVA with Dunnett’s multiple comparisons test) when compared to wt (see Table 4 for details).

**Fig 3:**
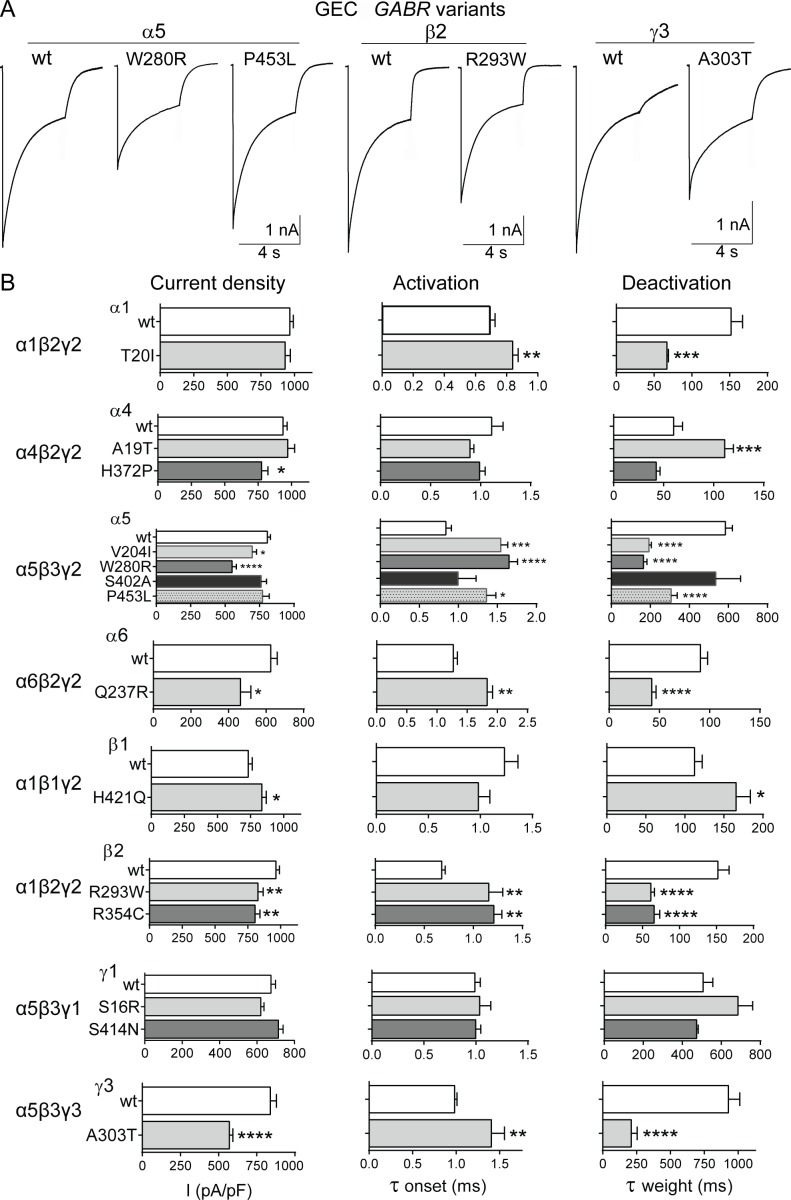
Deleterious GEC *GABR* variants caused major reductions of GABA-evoked currents. (A), Representative current traces from wild type (wt) and variant subunit-containing receptors are shown. The variants α5W280R, α5P453L, β2R293W, and γ3A303T were scored as deleterious by PolyPhen-2. GABA-evoked currents are the result of 4 s pulses of 1 mM GABA of cells expressing α5β3γ2, α1β2γ2, and α5β3γ3 GABA_A_ receptors, respectively. (B), Bar plots summarize the effects of wild type and GEC subunit variants on macroscopic parameters of GABA_A_ receptor currents. GABA_A_ receptor composition is indicated on the left. Values were expressed as mean ± S.E.M. *, **, *** and **** correspond to p < 0.05, p < 0.01, p < 0.001 and p < 0.0001 (unpaired t test or one-way ANOVA with Dunnett’s multiple comparisons test) statistically different from wild type (see Table 5 for details).

Effects on GABA_A_ receptor function were measured using a rapid exchange system to apply 1 mM GABA for 4 s to lifted HEK293T cells co-expressing wild type or variant subunits using 8 different GABA_A_ receptor subunit combinations (see Methods section). We determined peak GABA-evoked current amplitudes from receptors expressed on the cell surface (agonist-evoked current density), zinc sensitivity, and macroscopic current kinetic properties (activation onset and deactivation weight of currents evoked by applying GABA for 10 ms) (Figs 2 and 3) (Tables 4 and 5). Among the variants analysed, 9 (α1H129Y, α1T441M, β3V200I, β3I448V, γ2L57F, α5W280R, β2R293W, β2R354C, γ3A303T) out of 10 scored as deleterious, were found to be associated with significant reduction of GABA_A_ receptor current density (p *=* 0.0189, Fisher’s exact test) (Table 3 and S3 Table). In addition, reduction in function was strongly associated with 14 (α1H129Y, α1T441M, β3V200I, β3I448V, γ2L57F, α5W280R, β2R293W, γ3A303T, α1P29S, β3R194Q, β3R221K, β3R238W, α5V204I, α6Q237R) out of 17 variants mapped within the N-terminal and transmembrane domains (p *=* 0.0036, Fisher’s exact test) (Table 3 and S4 Table).

**Table 4:** Effects of ESP variants on the macroscopic properties of α1β3γ2 GABA_A_ receptors. Values are expressed as mean ± S.E.M.

Gene	Variant	Current Density(pA/pF)	Zinc Inhibition (%)	Activation onset (ms)	Deactivation Weight (ms)
*GABRA1*	α1D9E	725.7 ± 31.07 (13)	10.1 ± 1.61 (18)	1.295 ± 0.175 (13)	100.6 ± 18.29 (13)
	α1P29S	526.5 ± 49.74**** (24)	7.50 ± 1.66 (24)	1.284 ± 0.107 (42)	124.3 ± 8.720*** (42)
	α1H129Y	594.4 ± 51.94**** (25)	8.47 ± 1.72 (25)	1.230 ± 0.079 (27)	113.2 ± 12.53 (27)
	α1R147W	951.1 ± 52.41 (18)	7.83 ± 1.17 (18)	1.495 ± 0.120** (22)	49.60 ± 5.639* (22)
	α1T371I	994.7 ± 121.0 (18)	10.4 ± 2.39 (15)	1.289 ± 0.168 (21)	87.25 ± 3.666 (21)
	α1D383N	822.6 ± 43.50 (22)	9.91 ± 1.59 (22)	0.749 ± 0.047 (22)	153.1 ± 14.01*** (22)
	α1P409S	639.8 ± 32.32** (19)	11.1 ± 1.32 (19)	1.050 ± 0.086 (19)	75.81 ± 16.08 (19)
	α1K410R	850.6 ± 56.94 (28)	11.3 ± 1.43 (28)	1.188 ± 0.082 (21)	85.38 ± 12.94 (21)
	α1T441M	411.7 ± 32.48**** (24)	7.40 ± 1.61 (20)	1.574 ± 0.109**** (34)	51.31 ± 6.190* (34)
	Wt	871.4 ± 24.46 (72)	8.96 ± 0.87 (63)	1.063 ± 0.047 (70)	83.78 ± 3.525 (70)
*ANOVA summary (9 comparisons)*		p < 0.0001	p = 0.54	p < 0.0001	p < 0.0001
*GABRB3*	β3R194Q	668.2 ± 34.23*** (28)	9.04 ± 1.07 (28)	1.214 ± 0.115 (24)	76.42 ± 13.11 (24)
	β3D197N	770.4 ± 20.50 (35)	11.1 ± 0.82 (35)	1.677 ± 0.093*** (20)	66.62 ± 5.434 (20)
	β3V200I	645.7 ± 63.47**** (26)	10.9 ± 1.65 (26)	1.624 ± 0.167*** (28)	120.0 ± 15.55* (28)
	β3R221K	650.1 ± 40.02**** (29)	10.6 ± 0.99 (29)	1.437 ± 0.108* (30)	76.38 ± 10.13 (30)
	β3R238W	701.5 ± 22.86** (31)	12.4 ± 0.85 (30)	1.376 ± 0.103 (29)	74.71 ± 8.772 (29)
	β3D387N	644.2 ± 49.07*** (18)	12.9 ± 1.44 (18)	1.474 ± 0.106 (16)	56.87 ± 8.348 (16)
	β3I448V	620.8 ± 50.50**** (30)	11.5 ± 1.54 (30)	1.257 ± 0.172 (22)	108.5 ±20.39 (22)
	Wt	873.6 ± 24.71 (71)	9.47 ± 0.86 (60)	1.067 ± 0.048 (69)	82.96 ± 3.478 (69)
*ANOVA summary (7 comparisons)*		p < 0.0001	p = 0.25	p < 0.0001	p = 0.0018
*GABRG2*	γ2L57F	627.7 ± 19.03**** (71)	9.32 ± 1.22 (27)	1.049 ± 0.080 (28)	74.17 ± 10.85 (28)
	γ2A402T	1132 ± 92.23**** (23)	7.21 ± 1.94 (23)	0.934 ± 0.042 (19)	90.70 ± 3.77 (19)
	Wt	885.1 ± 21.82 (101)	9.14 ± 1.22 (92)	1.056 ± 0.047 (69)	83.68 ± 3.58 (69)
*ANOVA summary (2 comparisons)*		p < 0.0001	p = 0.48	p = 0.451	p = 0.30

*, **, *** and **** represent p < 0.05, p < 0.01, p < 0.001 and p < 0.0001 statistically different from wt after analysis of number of comparisons per gene by using one-way ANOVA with Dunnett’s multiple comparisons test. *n =* parenthesis. ESP = Exome Sequencing Project.

**Table 5:** Effects of GEC variants on the macroscopic properties of αβγ GABA_A_ receptors. Values are expressed as mean ± S.E.M.

Gene/Receptor	Variant	Current Density(pA/pF)	Zinc Inhibition (%)	Activation onset (ms)	Deactivation Weight (ms)
*GABRA1*/α1β2γ2	α1T20I	930.7 ± 38.53 (24)	9.00 ± 0.56 (18)	0.839 ± 0.034** (13)	67.10 ± 1.825*** (13)
	wt	965.0 ± 26.27 (38)	8.79 ± 1.05 (16)	0.690 ± 0.036 (19)	148.1 ± 15.53 (19)
*unpaired t test summary*		p = 0.45	p = 0.85	p = 0.0075	p = 0.0002
*GABRA4*/α4β2γ2	α4A19T	971.5 ± 51.89 (14)	35.8 ± 2.59 (10)	0.894 ± 0.040 (12)	111.0 ± 8.752*** (12)
	α4H372p	776.2 ± 48.04* (11)	23.0 ± 1.92 (11)	0.989 ± 0.056 (11)	42.63 ± 3.774 (11)
	wt	936.0 ± 31.85 (42)	28.5 ± 3.58 (12)	1.111 ± 0.114 (12)	59.92 ± 8.928 (12)
*ANOVA summary (2 comparisons)*		p = 0.035	p = 0.016	p = 0.17	p < 0.0001
*GABRA5*/α5β3γ2	α5V204I	697.0 ± 31.44* (49)	11.4 ± 2.05 (25)	1.544 ± 0.088*** (26)	192.9 ± 12.44**** (26)
	α5W280R	550.5 ± 31.69**** (41)	18.5 ± 2.55*** (39)	1.650 ± 0.109**** (37)	164.6 ± 18.18**** (37)
	α5S402A	761.7 ± 39.80 (15)	13.6 ± 1.17 (27)	0.996 ± 0.232 (12)	535.2 ± 127.4 (12)
	α5P453L	772.3 ± 48.55 (32)	13.9 ± 1.71 (18)	1.361 ± 0.119* (15)	306.8 ± 31.00**** (14)
	wt	806.7 ± 22.75 (40)	10.1 ± 0.68 (45)	0.840 ± 0.070 (20)	584.7 ± 35.98 (20)
*ANOVA summary (4 comparisons)*		p < 0.0001	p = 0.0044	p < 0.0001	p < 0.0001
*GABRA6*/α6β2γ2	α6Q237R	463.8 ± 55.61* (15)	35.6 ± 3.18* (10)	1.831 ± 0.091** (10)	42.44 ± 4.267**** (11)
	wt	625.0 ± 34.54 (10)	26.3 ± 2.11 (10)	1.270 ± 0.068 (10)	90.80 ± 7.084 (10)
*unpaired t test summary*		p = 0.040	p = 0.025	p = 0.0014	p < 0.0001
*GABRB1*/α1β1γ2	β1H421Q	835.5 ± 33.55* (21)	8.38 ± 1.43 (21)	0.981 ± 0.111 (18)	165.7 ± 18.29* (18)
	wt	732.9 ± 30.48 (32)	8.75 ± 1.13 (16)	1.231 ± 0.131 (11)	112.4 ± 9.863 (11)
*unpaired t test summary*		p = 0.032	p = 0.85	p = 0.16	p = 0.040
*GABRB2*/α1β2γ2	β2R293W	827.1 ± 39.68** (32)	10.8 ± 1.43 (16)	1.158 ± 0.143** (22)	61.23 ± 4.884**** (22)
	β2R354C	805.6 ± 36.89** (32)	8.90 ± 1.36 (18)	1.209 ± 0.082** (13)	65.72 ± 7.442**** (13)
	wt	965.0 ± 26.27 (38)	8.79 ± 1.05 (16)	0.678 ± 0.036 (20)	151.8 ± 15.20 (20)
*ANOVA summary (2 comparisons)*		p = 0.0017	p = 0.483	p = 0.0013	p < 0.0001
*GABRG1*/α1β2γ1	γ1S16R	619.8 ± 17.92 (15)	22.6 ± 2.22 (17)	1.036 ± 0.106 (13)	685.3 ± 76.15 (13)
	γ1S414N	714.2 ± 25.73 (16)	22.3 ± 1.76 (14)	0.998 ± 0.048 (10)	473.9 ± 8.257 (10)
	wt	674.9 ± 23.81 (24)	22.8 ± 1.56 (28)	0.990 ± 0.052 (14)	507.2 ± 49.47 (14)
*ANOVA summary (2 comparisons)*		p = 0.043	p = 0.98	p = 0.90	p = 0.034
*GABRG3*/α5β3γ3	γ3A303T	570.7 ± 22.45**** (20)	25.8 ± 2.80 (25)	1.405 ± 0.149** (10)	211.0 ± 43.19**** (12)
	wt	838.6 ± 40.05 (15)	29.6 ± 2.39 (21)	0.982 ± 0.027 (13)	928.4 ± 83.60 (13)
*unpaired t test summary*		p < 0.0001	p = 0.32	p = 0.0048	p < 0.0001

*, **, *** and **** correspond to p < 0.05, p < 0.01, p < 0.001 and p < 0.0001 statistically different from wild type (wt) after analysis of number of comparisons per gene by using one-way ANOVA with Dunnett’s multiple comparisons test. Unpaired *t* test (two-tailed) was used for single comparisons. *n =* parenthesis. GEC = genetic epilepsy case.

To address if changes in current density reflect the effects of receptor subunit composition and/or macroscopic current kinetic properties due to the presence of a variant, we first measured the sensitivity of GABA_A_ receptors to zinc inhibition, which depends on subunit composition (i.e. binary αβ receptors have high and ternary αβγ receptors have low zinc sensitivity) [31, 32], and then we compared the differences in wild type and variant partnering subunits on surface and total expression levels. Despite the significant reduction in current density, ESP variants showed no changes in sensitivity to blockade by zinc (Table 4), whereas among GEC variants, slight changes in zinc inhibition were found for only α5(W280R)β3γ2 and α6(Q237R)β2γ2 receptors (Table 5).

**Fig 4:**
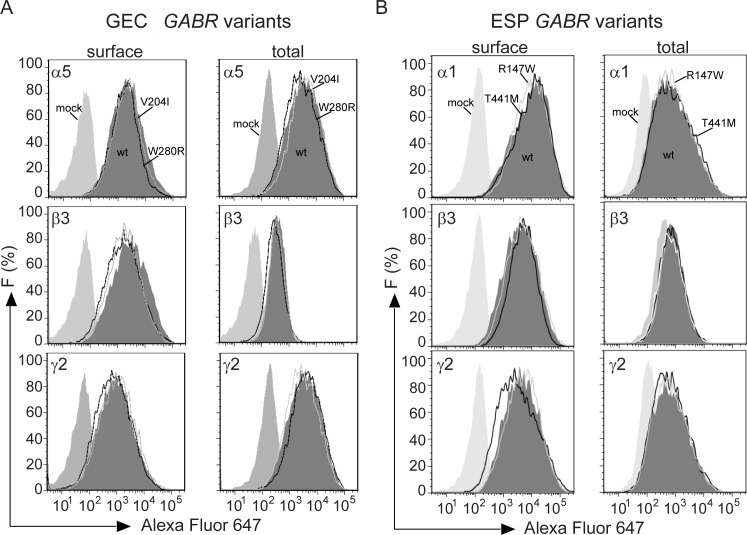
Deleterious *GABR* variants did not reduce surface and total levels of GABA_A_ receptor subunits. (A), Fluorescence intensity (F) of representative surface and total expression histograms of α5^HA^, β3, and γ2^FLAG^ subunits for wild type (wt) and α5β3γ2 receptors containing α5^HA^V204I and α5^HA^W280R GEC variants. Grey areas under the histograms represent mock (light) and wild type subunits (dark) respectively, while solid lines represent α5^HA^V204I (grey) and α5^HA^W280R (black) subunits. (B), Fluorescence intensity (F) of representative surface and total expression histograms of α1, β3, and γ2^HA^ subunits for wild type (wt) and α1β3γ2 receptors containing α1R147W and α1T441M ESP variants. Grey areas under the histograms represent mock (light) and wild type subunits (dark), respectively. Solid lines represent α1R147W (grey) and α1T441M (black) subunits.

Since significant reduction of GABA_A_ receptor function was strongly associated with deleterious variants that were mapped within the N-terminal and transmembrane domains, we used flow cytometry to asses if this was a consequence of reduced surface and total expression of receptor subunits. To determine this, we co-transfected HEK293T cells with wild type and variant α1, β1, β2, β3 and γ2^HA^ subunits for α1β(1, 2, 3)γ2 receptors, α(4, 5, 6)^HA^, β2, β3 and γ2^FLAG^ subunits for α(4, 5, 6)β(2, 3)γ2 receptors, and α5, α5^HA^, β3, γ(1, 3) and γ(1, 3)^HA^ subunits for α5β3γ(1, 3) receptors. Fig 4A shows the expression results for co-expressed β3, γ2L^FLAG^ and either wild type α5^HA^ subunit, α5^HA^V204I or α5^HA^W280R GEC variants in HEK293T cells. The α5^HA^W280R subunit expression levels were slightly, but not significantly, reduced compared to wild type α5^HA^ subunit levels, without significant reduction in α5^HA^V204I, β3 or γ2L^FLAG^ subunit levels (Fig 4A and S1 Table). Total expression levels of α5^HA^, α5^HA^V204I, α5^HA^W280R, β3 or γ2L^FLAG^ subunits remained unchanged. Fig 4B shows the expression results for co-expressed β3, γ2L^HA^ and either wild type α1 or α1R147W and α1T441M ESP in HEK293T cells. Following the same trend, the surface expression levels of α1R147W or α1T441M were not affected as well as for the β3 and γ2L^HA^ subunits (Fig 4B and S2 Table). No changes were also found for total expression levels of α1, α1R147W, α1T441M, β3 or γ2L^HA^ subunits. None of the variants reduced total or surface levels of α1, α4, α5, α6, β1, β2, β3, γ1, γ2, or γ3 subunits (S1 and S2 Tables). These results suggest that none of the *GABR* variants impaired GABA_A_ receptor synthesis or trafficking of wild type partnering subunits and were incorporated and expressed as pentameric αβγ receptors on the cell surface.

### Deleterious *GABR* rare variants that reduced GABA_A_ receptor gating caused structural perturbations along the β+/α- GABA binding interface

**Fig 5:**
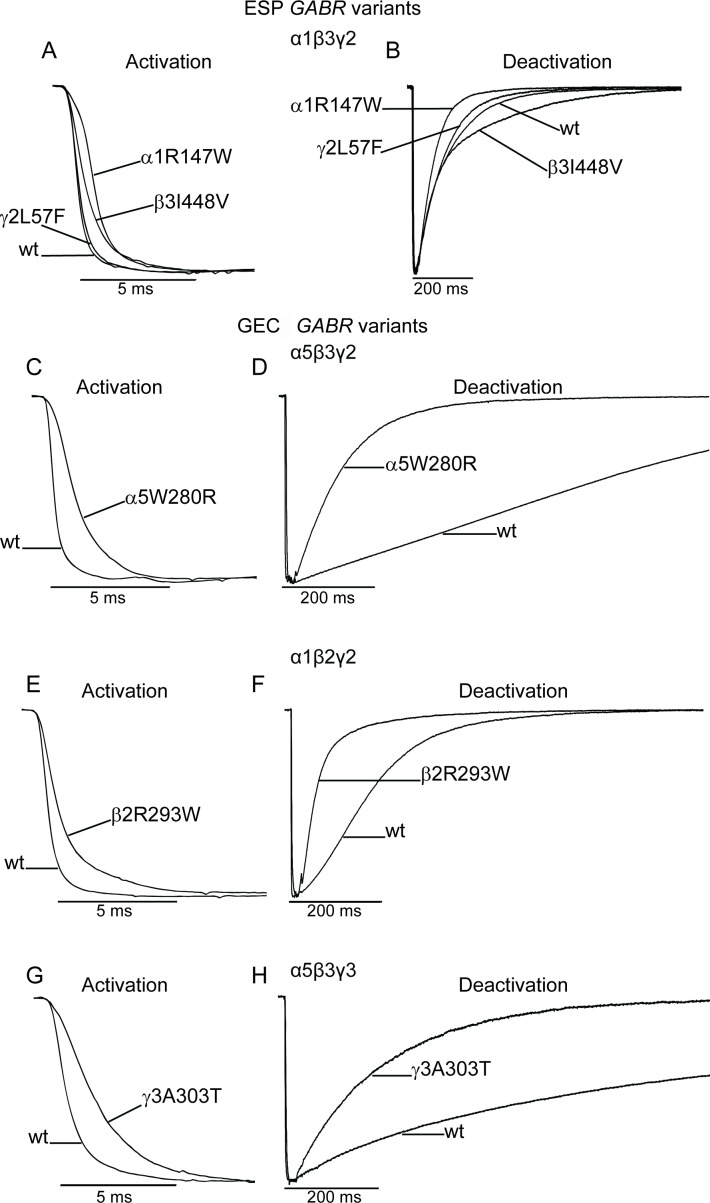
Deleterious *GABR* variants altered the kinetic properties of GABA-evoked currents. Representative current traces showing activation (A, C, E, G) and deactivation (B, D, F, H) properties of wild type (wt) and variant subunit-containing receptors were obtained following rapid application of GABA (1 mM, 10 ms). (A, B) GABA-evoked currents from cells expressing wild type α1β3γ2 GABA_A_ receptors and α1β3γ2 receptors containing α1R147W, β3I448V, and γ2L57F, which were all scored as deleterious by PolyPhen-2. (C-H) GABA-evoked currents from cells expressing α5β3γ2 (C and D), α1β2γ2 (E and F), and α5β3γ3 (G and H) wild type and variant GABA_A_ receptors. The variants α5W280R (C, D), β2R293W (E, F), and γ3A303T (G, H) were all scored as deleterious by PolyPhen-2. Traces are normalized for clarity.

To gain insights into whether *GABR* variants altered the kinetic properties of GABA_A_ receptors, we examined the activation and deactivation rates of macroscopic currents; properties that correlate with the average time channels are bound with GABA. We hypothesized that activation and deactivation are inversely correlated. Consequently, variant-bearing receptors with currents that have faster activation and prolonged deactivation would increase gating, while variant receptors with slower activation and accelerated deactivation would decrease gating. We determined current activation and deactivation by measuring the time constant (τ) of current onset (Fig 5A, 5C, 5E and 5G) and offset (Fig 5B, 5D, 5F and 5H) during and following GABA (1 mM, 10 ms) stimulation (Tables 4 and 5). ESP variants either slowed or had no effect on activation; while deactivation was either not affected or altered in opposite directions (prolonged or accelerated) (Fig 5A and 5B). Conversely, most GEC variants slowed activation and accelerated deactivation (Fig 5C–5H).

**Fig 6:**
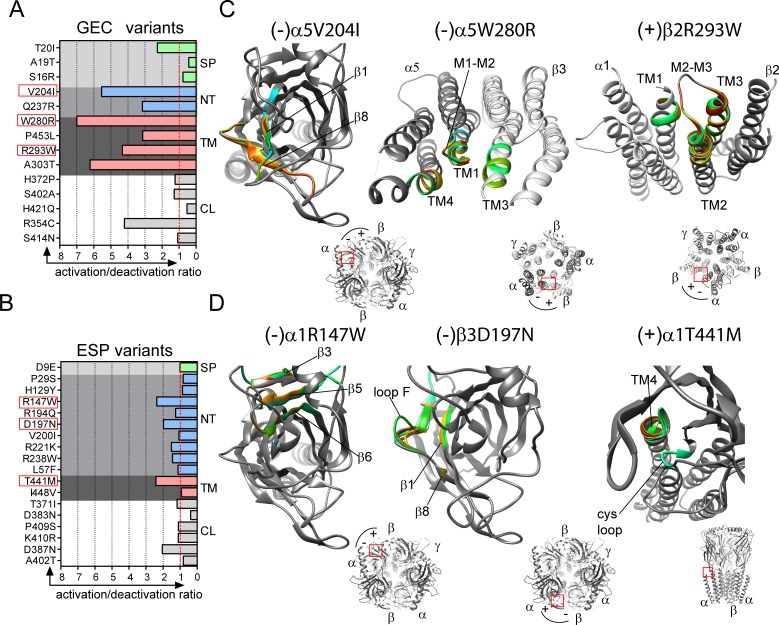
Gating defective *GABR* variants were mapped to β-strands and transmembrane domains of the GABA_A_ receptor. (A, B) Gating impairment ratio plots for GEC and ESP variants were classified by GABA_A_ receptor structural domains. Impairment ratio equal to 1 (red dashed lines) indicate no change in gating. GEC and ESP variants shown in panels (C) and (D) are highlighted by red boxes. (C, D) Enlarged views of the domains that have structural rearrangements caused by GEC (-)α5V204I, (-)α5W280R, (+)β2R293W, ESP (-)α1R147W, (-)β3D197N, and (+)α1T441M. The structural elements that differ among wild type and variant structures are indicated by solid black lines and are depicted in grey (wild type) and rainbow (variant, RMS ≥ 0.5 Å). (+) and (-) indicate the interface where the variant subunit was mapped. The lower left panels show the localization of the variants (red boxes) in the 3-D GABA_A_ receptor as seen in Fig 1. Signal peptide (SP), NT (N-terminal), TM (transmembrane) and M3/M4 cytoplasmic loop (CL).

Based on our functional results, we computed gating impairment as the ratio of activation/deactivation time constants after 10 ms GABA stimulation (Fig 6A and 6B and Tables 4 and 5). Because macroscopic activation and deactivation are coupled during gating [33], when the receptor undergoes conformational changes between open and closed states, the resulting ratio is an estimate of decreased or increased gating resulting from the variant. We found that slow activation and fast deactivation produced significant gating impairment (ratio > 1) and fast activation and slow deactivation resulted in small gating impairment (ratio ≤ 1). It was striking that 12 (α5V204I, α6Q237R, α5W280R, α5P453L, β2R293W, γ3A303T, α1R147W, β3R194Q, β3D197N, β3R221K, β3R238W, α1T441M) of 17 variants mapped within the N-terminal and transmembrane domains were found to be strongly associated with reduction of receptor gating (p *=* 0.0060, Fisher’s exact test) (S5 Table); and the remaining 5 (α1H129Y, β3V200I, β3I448V, α1P29S, γ2L57F) of 17 variants had no effect. In contrast, 12 (α4H372P, α1P409S, α4A19T, α5S402A, β1H421Q, γ1S16R, γ1S414N, α1D9E, α1T371I, α1D383N, α1K410R, γ2A402T) of the 15 variants mapped within the signal peptide and cytoplasmic loop were found to have a negligible effect on gating (S5 Table) and the remaining 3 (α1T20I, β2R354C, β3D387N) variants produced gating defect.

**Fig 7:**
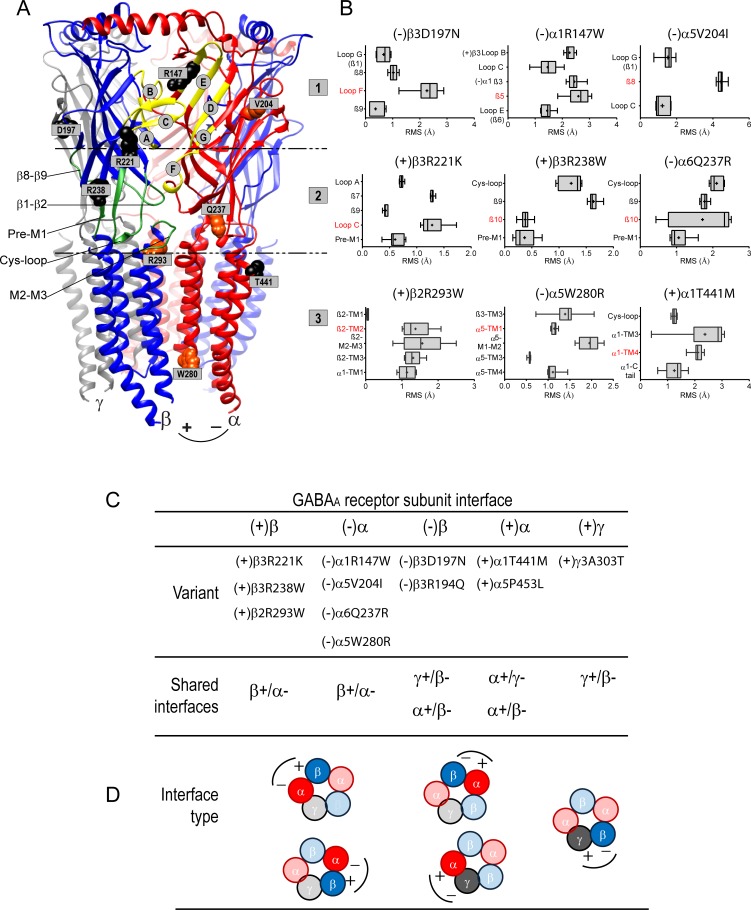
Structural perturbations caused by *GABR* variants were mapped along domains that couple agonist binding to channel opening. (A), A 3-D structural model of the GABA_A_ receptor shown from the side with important functional domains delimited by traced lines: the binding site (1), the coupling interface (2), and the transmembrane domain (3). The binding site is formed by the convergence of loops A-C from β(+) and loops D-G from α(-) at the β+/α- interface, as indicated. The coupling interface is formed by the β1-β2 and Cys-loops, the pre-M1 domain, the β8-β9 loop and the M2-M3 linker, as indicated. *GABR* variants were mapped on the structure, and are represented in orange and black for GEC and ESP, respectively. (B), Box plots show perturbations (RMS deviation) in side chain residues of β-strands, loops and TM helices caused by the different variants. RMS deviations for 10 simulations are represented as interleaved box and whiskers (25–75% percentile, median, minimum and maximum, and mean as +) by structural element. The secondary structure containing the mutation is highlighted in red. Top panels represent subunit variants mapped to the binding site, middle panels in the coupling interface, and lower panels in the transmembrane domain. Panel C lists the location of the variants and theirs respective interfaces. Variants were classified according to the type of shared interface. (D), Cartoon representation of the subunit arrangement of GABA_A_ receptors containing variants in α, β, or γ subunits (darker subunits). Principal (+) and complementary (-) interfaces of each subunit shown.

Taking into account that the variants are located at or near the β+/α-, α+/β-, α+/γ- or γ+/β- interfaces in receptor domains that contribute to the transition mechanics from the shut to the open state, we propose that these mutations might affect receptor structure in different ways. To assess the specific contribution of the individual variants on the structural coupling mechanism, we performed wild type and variant pentameric α5β3γ2, α1β2γ2, α1β3γ2, and α6β2γ2 GABA_A_ receptor simulations using the solved structure of GluClα or the human GABA_A_R-β3 as template. We simulated 15 of the 17 variants mapped to the principal ((+)α, (+)β) and complementary ((-)α, (-)β, (-)γ) interfaces of the α, β and γ subunits (Figs 6 and 7 and S1 and S2 Figs). The major structural changes induced by 10 ((-)α5V204I, (-)α5W280R, (-)α6Q237R, (+)β2R293W, (-)α1R147W, (+)α1T441M, (-)β3R194Q, (-)β3D197N, (+)β3R221K, (+)β3R238W) gating defective variants occurred at the interface between the principal side of the β subunit and the complementary side of the α subunit (β+/α- interface) (Figs 6C and 6D and 7 and S1 Fig). We evaluated the structural rearrangements of the subunit secondary structure and side chain conformational changes that might occur by computing the root mean square (RMS) deviation. This method enabled comparison of disturbances produced by the presence of the variant in the structure for any domain. Consequently, the larger the RMS deviation (≥ 0.5 Å), the more the mutant structure deviates from the wild type structure. Fig 6 highlights some examples of gating defective GEC (-)α5V204I, (-)α5W280R, (+)β2R293W and ESP (-)α1R147W, (-)β3D197N, (+)α1T441M variants causing a wave of propagated structural adjustments among loops, β-strands and transmembrane domains (Fig 6C and 6D, 3-D structures: wild type in grey, variant-associated alternative secondary structural conformations in rainbow when RMS > 0.5 Å).

We predicted perturbations of the secondary structure and side chain residues through neighbouring structural domains at the β+/α-, γ+/β-, α+/β-, and α+/γ- interfaces for both GEC and ESP variants (Fig 7 and S1 Fig). These structural rearrangements were local when confined to structural domains of the affected subunit ((-)α5V204I, (-)β3R194Q, (-)β3D197N, (+)β3R221K, (+)β3R238W, (-)α6Q237R, (+)α1T441M), and global when propagated to the nearest subunit through rearrangements of nearby structural domains ((-)α1R147W, (-)α5W280R, (+)β2R293W). We identified that the rearrangements were restricted to three distinct functional domains in the GABA_A_ receptor: binding site, coupling interface, and transmembrane domain (Fig 7A and 7B).

At the β+/α- interface, the (-)α1R147W and (-)α5V204I variants caused side chain perturbations (RMS > 1–5 Å) in loops B ((+)β3), C ((+)β3), E ((-)α1) and G ((-)α5) that form the ligand-binding site. The (+)β3R221K variant propagated rearrangements (RMS > 0.5–2 Å) from loops A and C in the binding site to the pre-M1 domain in the coupling interface, while side chain perturbations (RMS > 0.5–2.5 Å) caused by (+)β3R238W and (-)α6Q237R variants were confined to the Cys-loop and the pre-M1 domain in the coupling interface.

In the transmembrane domain, (-)α5W280R and (+)β2R293W variants caused local ((-)α5-TM1, (-)α5-M1-M2 loop, (-)α5-TM4, (+)β2-TM2, (+)β2-M2-M3-loop, (+)β2-TM3) and global ((+)β3-TM3, (-)α1-TM1) rearrangements (RMS > 0.5–2 Å) that link the coupling interface to the transmembrane domain. Conversely, (-)β3R194Q, (-)β3D197N and (+)α1T441M variants propagated rearrangements (RMS > 0.5–3 Å) from the binding site (loops G and F, β8-strand) to the coupling interface (Cys-loop) at the γ+/β-, α+/β-, and α+/γ- interfaces. Despite being opposite to the main interface, the interface convergence form homologous functional domains can impair receptor gating as reported previously [34–37]. In contrast, ESP (-)β3V200I, (-)β3I448V, (+)α1H129Y, (-)α1P29S and (-)γ2L57F variants that had no effects on channel gating produced perturbations that were mainly located on complementary GABA_A_ receptor subunit interfaces (α+/β-, α+/γ-, and γ+/β-) (S2 Fig). Overall, variants mapped along the β+/α- GABA binding interface were strongly associated with reductions in channel gating and were predicted to cause receptor structural rearrangements.

### GEC *GABR* variants reduced GABA potency

**Fig 8:**
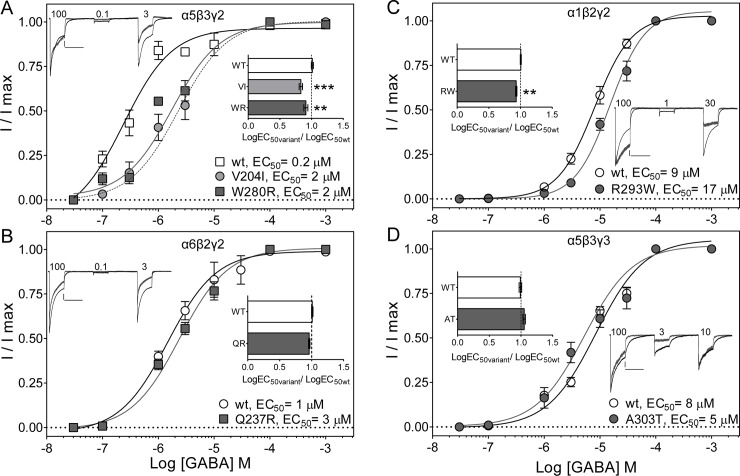
Gating defective GEC *GABR* variants caused right-shifts on GABA concentration-response curves. (A) GABA concentration-response curves for α5V204I, and α5W280R, (B) α6Q237R, (C) β2R293W, and (D) γ3A303T subunit variants (filled symbols) and wild type (wt) receptors (open symbols) were normalized to the maximum current evoked by a saturating agonist concentration. GABA_A_ receptor composition is noted for each panel. Inside the panels, superimposed peak currents evoked by a 4 s application of various concentrations of GABA (μM) are shown; as indicated for wild type (in black) and α5W280R (A), α6Q237R (B), β2R293W (C), and γ3A303T (D) variant receptors (grey). Amplitude scale bars for wild type and variant current traces are 500/75 pA (A), 500/200 pA (B), 500/100 pA (C), and 200/50 pA (D), respectively. All time scale bars represent 5 s. Insert depicts the LogEC_50_ variant/LogEC_50_ wild type ratio showing no significant variation among wild type receptors (A, 1.01 ± 0.02, *n =* 8; B, 1.01 ± 0.01, *n =* 5; C, 1.00 ± 0.01, *n =* 5; and D, 0.99 ± 0.02, *n =* 5). Values are expressed as mean ± S.E.M (see text for variant values). ** and *** indicate p < 0.01 and p < 0.001 statistically different from wild type (wt) after analysis by one-way ANOVA with Dunnett’s multiple comparisons test for panel A (p *=* 0.0002). Unpaired t test (two-tailed) was used for single comparisons for panels B to D (p *=* 0.0558, p *=* 0.0011, p *=* 0.0667, respectively).

To determine whether changes in gating cause measurable changes in GABA_A_ receptor potency, we measured the effects of variants on GABA concentration-response curves (Fig 8). Consequently, we studied a group of GEC variants associated with major reductions in gating and located in between the ligand-binding and pore-forming domains (α5V204I, α6Q237R, α5W280R, β2R293W, γ3A303T) (Figs 5C–5H and 7). Macroscopic peak currents were evoked by applying various concentrations of GABA for 4 s to wild type and variant α5β3γ2, α6β2γ2, α1β2γ2, and α5β3γ3 GABA_A_ receptors.

We confirmed that subunit composition greatly influences how GABA_A_ receptors responded to GABA. Wild type α5β3γ2 receptors displayed the highest potency for GABA (EC_50_ = 0.2 μM), followed by α6β2γ2, α1β2γ2 and α5β3γ3 receptors (EC_50_ for α5β3γ2 < α6β2γ2 < α1β2γ2 = α5β3γ3). For any given subunit variant-containing GABA_A_ receptor, maximum GABA responses were normalized by wild-type response for all GABA concentrations used. α5V204Iβ3γ2 and α5W280Rβ3γ2 variant receptors had a 10-fold increase in agonist EC_50_ (Fig 8A). α1β2R293Wγ2 caused a slight, but significant, 2-fold increase in EC_50_, and no effect was observed on α6Q237Rβ2γ2 and α5β3γ3A303T receptors (Fig 8B–8D).

The reduced GABA potency observed on the α5V204I, α5W280R, and β2R293W variants was confirmed by comparing the variant/wild type LogEC_50_ change ratio, which defines the gain- (ratio > 1) or loss- (ratio < 1) of-function caused by the mutation. α5V204Iβ3γ2, α5W280Rβ3γ2, and α1β2R293Wγ2 receptors displayed ratios less than 1 (0.83 ± 0.02, *n =* 5; 0.90 ± 0.03, *n =* 5; 0.93 ± 0.01, *n =* 5, respectively). Inversely, α6Q237Rβ2γ2 and α5β3γ3A303T variant receptors (0.96 ± 0.01, *n =* 5; 1.05 ± 0.02, *n =* 5, respectively) had minor or no effect on the ratios. Therefore, the gating defects caused by GEC α5V204I, α5W280R and β2R293W led to a reduction in GABA potency.

## Discussion

Molecular characterization of the contribution of the *GABRs* as susceptibility alleles for GEs is missing. Since an important role of GABA_A_ receptors is maintaining inhibitory tone in the brain, the identification of hundreds of variants in epilepsy and non-epilepsy subjects through whole exome sequencing studies suggests that they are likely to be pathogenic. In the present work, we investigated the effects of thirty-two *GABR* variants on receptor function and biogenesis. We found that both groups of variants decreased macroscopic current amplitudes by decreasing channel gating without reducing receptor surface expression. Furthermore, structural modelling predicted variant-induced rearrangements of inter- and intra-subunit secondary structures and side chains that may underlie channel kinetic defects, thus leading to disinhibition and GEs.

A major finding of this study was that the variants caused coupled structural-gating deficiencies of the receptor. Twelve variants were mapped to β-strands and transmembrane domains, and seven along the β+/α- GABA binding interface (Fig 7). Our structural simulations confirmed that the conformational changes occurred mainly in the primary functional domains of the receptor, the extracellular N-terminal domain of the receptor that contains the GABA binding site and the coupling interface, and the transmembrane domain that contains the ion channel [33, 36, 38–41]. Gating deficiencies were greater for variants located in the binding domain than in the coupling interface (Fig 6). The largest effect, however, was observed for variants positioned in the transmembrane domain, where the channel pore and channel gate are located.

Taking into account that the receptor has five subunit-subunit interfaces, two β+/α- interfaces, and single γ+/β-, α+/β- and α+/γ- interfaces (Fig 1), the effect of the variant on receptor function will be influenced by receptor stoichiometry (Fig 7C and 7D). If a heterozygous variant is present that codes for a residue in a (+)β or (-)α subunit interface region, zero, one or two β+/α- interfaces would be affected in the variant receptor. When a heterozygous variant is present that codes for a residue in an (-)β or (+)α subunit interface region, only zero or one of two different interfaces would be affected in the variant receptor. Conversely, when a heterozygous variant is present that codes for a residue in an (+)γ or (-)γ subunit interface region, only zero or one of a single interface would be affected in the variant receptor. Thus, the variants can affect receptor function by their subunit number and location in the pentamer as well as their functional domain in the receptor.

We suggest that the degree of channel dysfunction depends on whether the variant is located in a specific subunit or by combination of several interfaces containing key structural domains. Consequently, the presence of variants occurring in the (+)β and (-)α interfaces caused larger defects in channel gating (Figs 6 and 7) than those with a distribution in the receptor mapped at the (-)β, (+)α and (+)γ subunit interfaces. Since the β subunit initiates the uncapping of loop C upon agonist binding [36, 38], structural perturbations at the site (local) or neighbouring residues (global) caused by variants/mutations predict greater defects in the gating of the channel. In line with this assumption, comparable changes in gating properties and lack of effects in cellular surface and total expression levels were described for *de novo GABRB* mutations associated with severe epileptic encephalopathies [12]. Thus, mutations with pronounced defects in GABA_A_ receptor function and mapped in the (+)β subunit interface ((+)β3D120N, (+)β3E180G, (+)β3Y302C), were associated with a different disease syndrome than those mutations in the (-)β subunit interface ((-)β3N110D and (-)β1F246S). It is noteworthy that structural simulations predicted rearrangements of loop B, loop C, Cys-loop and the M2-M3 loop for those mutations in the (+)β subunit interface (see Figs 6 and 7 for comparison with *GABR* variants), which compromise the GABA binding pocket and the binding-coupling pathway to the transmembrane domains [33, 39]. Furthermore, *GABRG2(R82Q)* and *GABRG2(K328M)* mutations that are mapped in the (+)γ subunit interface mutations were associated with mild epilepsy syndromes [16, 42] and were found to disrupt GABA_A_ receptor function differentially [11]. In comparison, (+)γ2K328M and (-)β1F246S mutations shared a common molecular mechanism that caused GABAergic disinhibition by gating deficiency. Both mutations predicted rearrangements at both γ+/β- and α+/β- interfaces and propagated structural perturbations through the Cys-loop and the M2-M3 loop to the proximal extracellular transmembrane domain M1, which is critical for fast desensitization–deactivation coupling among all GABA_A_ receptor subunits [33]. Like the (+)β3Y302C mutation [12], the *GABRB2(M79T)* mutation associated with intellectual disability and epilepsy [19] which lies in the β2-sheet at the β+/α- interface, is expected to cause rearrangements within the Cys-loop and M2-M3 loop. In addition, variants of the structurally related glycine and acetylcholine Cys-loop receptors mapped in the binding-coupling pathway were found to impair the gating of the receptor [43–46]. Thus, our data are in accord with the β+/α- interface as being physically important for coupling agonist binding to receptor gating, and demonstrate that there is a structure-dysfunction correlation associated with the location of the mutation on the receptor, which is conserved among Cys-loop receptors.

*GABR* variants reduced gating via accelerated current deactivation, slowed activation, and yielded smaller current amplitudes, all changes that lead to reduced peak amplitude and altered time course of synaptic receptor-mediated inhibitory post-synaptic currents and promote hyperexcitability. Because these receptors regulate excitability at postsynaptic sites, it is likely that deficits in any subunit cause compensatory changes in other subunits. Since GABA_A_ receptor subunits display a distinctive distribution within the thalamocortical circuitry, the defects in function of the channel caused by the presence of variants result in reduction in GABA-mediated inhibition [47–50]. A compensatory mechanism may result in relative changes in both phasic and tonic inhibition. In support of this, genetic and pharmacological models of absence epilepsy showed increased activation of extra-synaptic GABA_A_ receptors and augmented tonic GABAergic inhibition in thalamocortical neurons [48]. In addition, experimental models of epilepsy showed that either decrease in α5 and δ subunit expression entailed paradoxical increase in tonic currents [51] or shifted the γ2 subunits to perisynaptic sites altering both tonic and phasic inhibitions [52].

Missense or nonsense mutations of *GABRA1*, *GABRB3* and *GABRG2* epilepsy genes are linked with classical GEs with autosomal dominant inheritance including, GEFS+, childhood absence epilepsy, febrile seizures, and juvenile myoclonic epilepsy. These mutations have been shown primarily to impair subunit biogenesis via degraded subunit mRNA or protein, reduced receptor assembly, endoplasmic reticulum retention of mutant receptors and subunits, subunit truncation with a dominant negative effect or impaired subunit oligomerization. Contrary to the above, the results from the present study suggest that the primary mechanism through which the *GABR* variants affected receptor function is via gating deficiencies and not via impaired biogenesis or receptor expression.

We found that variants in the epilepsy-associated genes *GABRA1*, *GABRB3* and *GABRG2* were mainly present in the ESP variants, but the GEC cohort contained epilepsy-associated *GABRA6*, *GABRB1*, and *GABRB2* and non-epilepsy- associated *GABRA4*, *GABRA5*, and *GABRG3* genes. Our results suggested that in the general population, variants in epilepsy genes reduce GABA_A_ receptor currents enough to likely confer risk for GEs, but not to cause GEs. Whereas in the reported group of GE patients, variants in non-epilepsy genes reduced GABA_A_ receptor currents to a similar extent and may act as modifiers of susceptibility in individuals predisposed to epilepsy. Complex or polygenic inheritance (involving two or more susceptibility genes) has been described in linkage analysis in childhood absence epilepsy families with a microdeletion of chromosome 15q13.3, which compromises the locus for *GABRG3*, *GABRA5* and *GABRB3*, and with rare variation of *GABRD* [53, 54]. These studies suggest that the presence of deleterious variation of epilepsy and non-epilepsy *GABR*s in a heterogeneous genetic background is most likely to contribute to reduction of seizure threshold in sporadic cases or small families.

The use of next-generation sequencing in genomic studies has contributed to the identification of numerous common and rare variants in subjects with family history of genetic epilepsy and in the general population. Combining *in vitro* and *in silico* proof of concept validations, our data provide strong functional evidence that deleterious variants may predict functional risk for loss of GABAergic function in individuals susceptible to epilepsy in the general population. Our study reveals the importance of GABA_A_ receptor function in maintaining central synaptic inhibition and reveals the pathophysiology of epilepsy from a standpoint that has not been considered before.

## Conclusions

- Sporadic genetic epilepsies (GEs) with predicted complex gene variant profiles are more difficult to interpret due to the poorly understood contribution of deleterious GABA_A_ receptor gene (*GABR)* variants present in both affected and unaffected individuals.
- We found that most *GABR* variants present in both affected and unaffected individuals reduced GABA_A_ receptor evoked currents with no changes in cellular surface or total expression levels, and thus the molecular mechanisms of variants underlying GABA_A_ receptor dysfunction was through reduced channel activation (binding / transduction / gating).
- The majority of *GABR* variants in the N-terminal and transmembrane domains, but not in the signal peptide or cytoplasmic domain, were scored as deleterious on the structure of the GABA_A_ receptor subunit and were mapped along the β+/α- interface that contains the GABA binding pocket.
- Our findings confirm the deleterious effects of GABR variants and highlight the contribution of GABA_A_ receptors to the pathogenesis of GEs.

## Supporting Information

S1 Fig GABA_A_ receptor β3 subunit variants with gating effects predicted similar structural rearrangements on the simulations when using two crystal models. Structural simulations for GABA_A_ receptor variants in the β3 subunit were built based on the human GABA_A_R-β3 (PDB: 4COF) and GluCl (PDB: 3RHW) crystal structures for comparison as referenced in the methods section. In A, root mean square (RMS) deviation bar plots (left panels) show disordered side chain residues through β-sheets and loops. In the right, structural β3 subunit simulations display the wild type in grey, and alternative secondary conformations in rainbow (ribbon). In B, structural β3 subunit simulations built using the 3RHW structure are shown here, and resultant RMS deviation bar plots are displayed in Fig 7. For the β3R194Q variant, β3 simulations using both 3RHW and 4COF crystal structures and RMS deviation bar plots are shown. RMS deviation values for up to 10 simulations are represented as interleaved box and whiskers by structural elements (25–75% percentile, median, minimum and maximum, and mean as +).
(TIF)

S2 Fig GABA_A_ receptor variants with no effects on gating predicted minor structural perturbations. Structural β3 simulations were compared on 3RHW (A) and 4COF (B) crystal structures as described in S1 Fig for variants β3V200I and β3I448V. RMS deviation bar plots (left panels) and β3 subunit simulations (right panels) are displayed with predicted perturbations by structural domains. Structural α1H129Y, α1P29S and γ2L57F simulations were built using the 3RHW (A) crystal structure. The α1H129Y simulation predicts perturbations that are mainly mediated through loops, whereas α1P29S and γ2L57F predict perturbations that are restricted to the α1-helix of the N-terminal domain (only RMS deviation bar plots are shown).
(TIF)

S1 Table No Effects of GEC variants on the expression properties of GABA_A_ receptors. (PDF)

S2 Table No Effects of ESP variants on the expression properties of α1β3γ2 GABA_A_ receptors. (PDF)

S3 Table Distribution of missense *GABR* variants by deleteriousness and effect on GABA-evoked currents. (PDF)

S4 Table Distribution of missense *GABR* variants by GABA_A_ receptor structural domains and GABA-evoked currents. (PDF)

S5 Table Distribution of missense *GABR* variants by GABA_A_ receptor structural domains and receptor gating. (PDF)
