# Correction: Deleterious Rare Variants Reveal Risk for Loss of GABA_A_ Receptor Function in Patients with Genetic Epilepsy and in the General Population

**DOI:** 10.1371/journal.pone.0167264

**Published:** 2016-11-21

**Authors:** Ciria C. Hernandez, Tara L. Klassen, Laurel G. Jackson, Katharine Gurba, Ningning Hu, Jeffrey L. Noebels, Robert L. Macdonald

In Table 2, three variants corresponding to the GABRP gene were incorrectly described. The R200H variant should be R200H/C, the S293P variant should be R293C, and the R389N variant should be D389N. Please see the corrected Table 2 here.

**Table 2:** Unique GABR variants from GECs reported in the 237 ion channel genes project^1^. ^1^GECs = genetic epilepsy cases [20]. *GABR variants characterized in this study.

*GABR* gene	Variant	Occurrence of variants among GECs
*GABRA1*	T20I*	1
*GABRA4*	H372P*	1
*GABRA5*	W280R*	3
*GABRA5*	P453L*	1
*GABRB2*	R293W*	1
*GABRG3*	A303T*	1
*GABRA4*	A19T*	1
*GABRA5*	V204I*	1
*GABRA5*	S402A*	1
*GABRA6*	Q237R*	1
*GABRB1*	H421Q*	1
*GABRB2*	R354C*	2
*GABRG1*	S16R*	1
*GABRG1*	S414N*	1
*GABRE*	R472H	1
*GABRE*	S484L	1
*GABRP*	R200H/C	2
*GABRP*	S292P	1
*GABRP*	R293C	1
*GABRP*	D389N	1
*GABRR2*	R287H	1
*GABRR2*	V294I	2
*GABRE*	R452G	1
*GABRP*	V349A	5
