# Supplementary figures and images for: Deleterious Rare Variants Reveal Risk for Loss of GABAA Receptor Function in Patients with Genetic Epilepsy and in the General Population
Source: PLoS One. 2016 Sep 13;11(9):e0162883. doi: 10.1371/journal.pone.0162883 (PMC5021343; doi:10.1371/journal.pone.0162883)

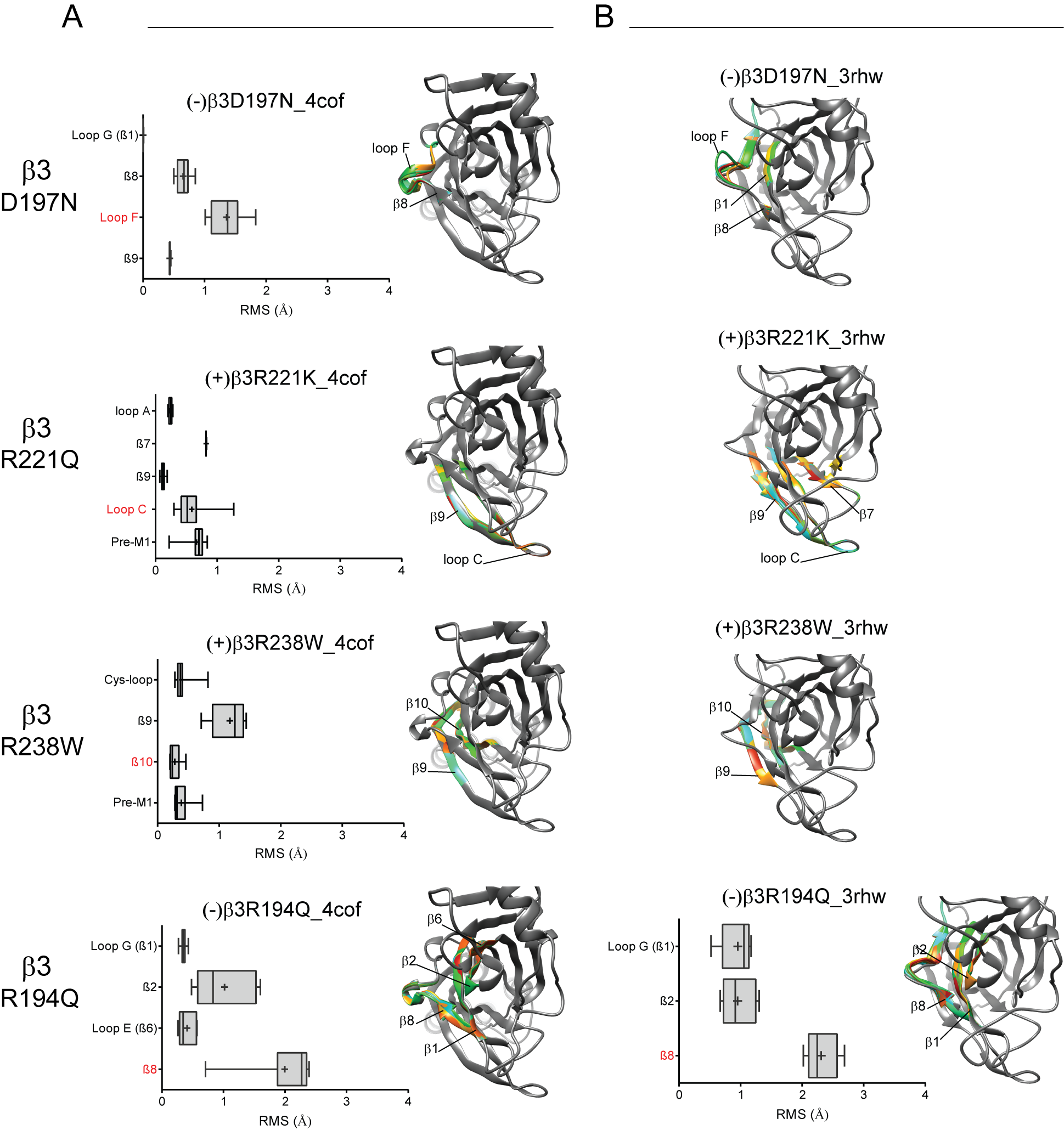

Supplement: S1 Fig — Structural simulations for GABAA receptor variants in the β3 subunit were built based on the human GABAAR-β3 (PDB: 4COF) and GluCl (PDB: 3RHW) crystal structures for comparison as referenced in the methods section. In A, root mean square (RMS) deviation bar plots (left panels) show disordered side chain residues through β-sheets and loops. In the right, structural β3 subunit simulations display the wild type in grey, and alternative secondary conformations in rainbow (ribbon). In B, structural β3 subunit simulations built using the 3RHW structure are shown here, and resultant RMS deviation bar plots are displayed in Fig 7. For the β3R194Q variant, β3 simulations using both 3RHW and 4COF crystal structures and RMS deviation bar plots are shown. RMS deviation values for up to 10 simulations are represented as interleaved box and whiskers by structural elements (25–75% percentile, median, minimum and maximum, and mean as +). (TIF) [file pone.0162883.s001.tif]

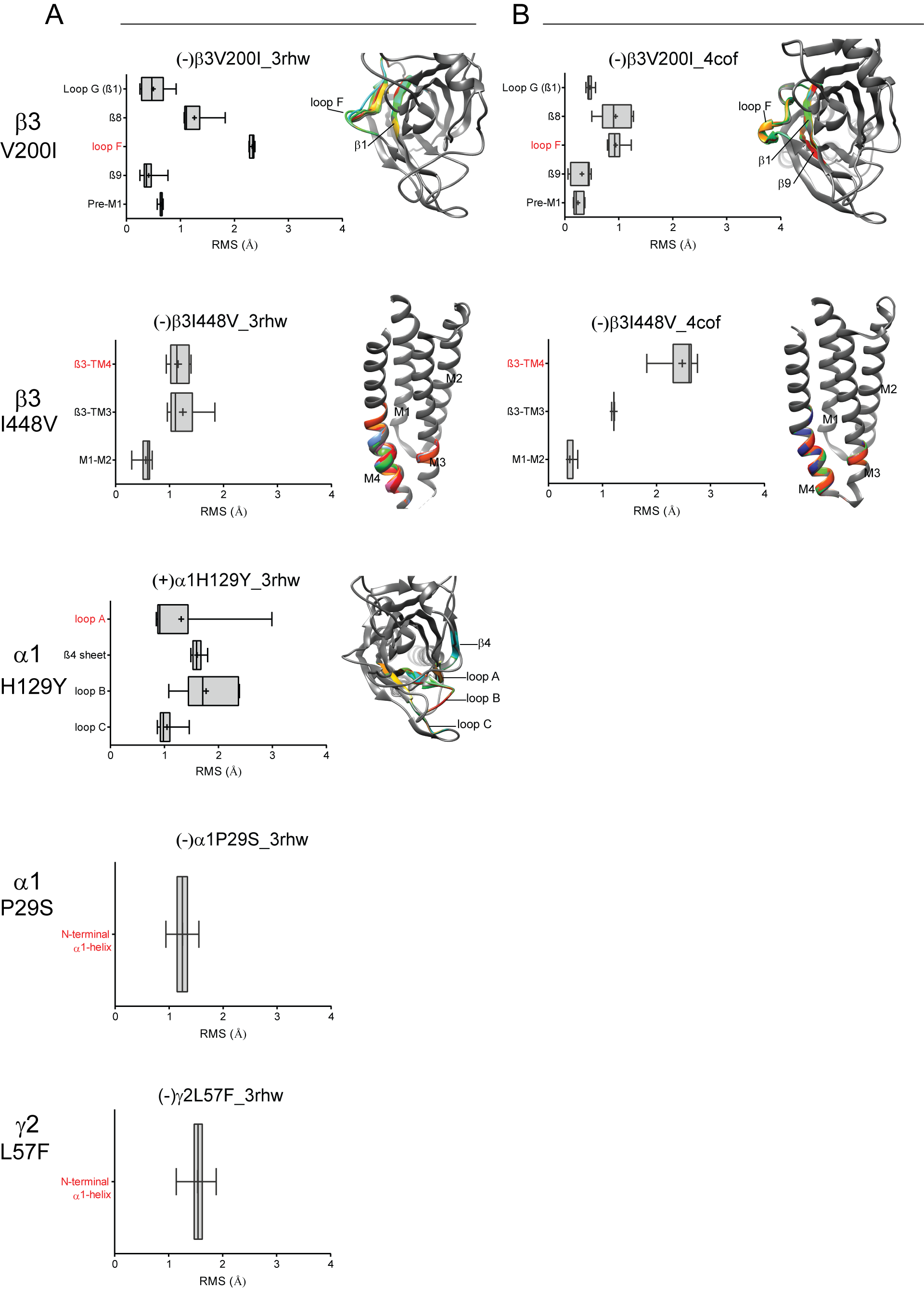

Supplement: S2 Fig — Structural β3 simulations were compared on 3RHW (A) and 4COF (B) crystal structures as described in S1 Fig for variants β3V200I and β3I448V. RMS deviation bar plots (left panels) and β3 subunit simulations (right panels) are displayed with predicted perturbations by structural domains. Structural α1H129Y, α1P29S and γ2L57F simulations were built using the 3RHW (A) crystal structure. The α1H129Y simulation predicts perturbations that are mainly mediated through loops, whereas α1P29S and γ2L57F predict perturbations that are restricted to the α1-helix of the N-terminal domain (only RMS deviation bar plots are shown). (TIF) [file pone.0162883.s002.tif]
