# Supplementary material for: Deleterious Rare Variants Reveal Risk for Loss of GABAA Receptor Function in Patients with Genetic Epilepsy and in the General Population
Source: PLoS One. 2016 Sep 13;11(9):e0162883. doi: 10.1371/journal.pone.0162883 (PMC5021343; doi:10.1371/journal.pone.0162883)
Supplement: S1 Table — (PDF) [file pone.0162883.s003.pdf]

S1 TABLE

No Effects of GEC variants on the expression properties of $GABA_A$ receptors

		Surface			Total		
Gene/ Receptor	Variant	α	β	γ	α	β	γ
GABRA1 / α1β2γ2	α1T20I	0.97 ± 0.02 (3)	1.21 ± 0.12 (3)	1.03 ± 0.08 (3)	1.02 ± 0.05 (3)	0.93 ± 0.14 (3)	1.00 ± 0.09 (3)
GABRA4 / α4β2γ2	α4A19T	1.16 ± 0.04 (3)	1.10 ± 0.07 (4)	0.82 ± 0.06 (4)	1.24 ± 0.09 (3)	1.06 ± 0.15 (3)	0.95 ± 0.03 (4)
	α4H372P	0.88 ± 0.02 (4)	0.96 ± 0.02 (3)	0.93 ± 0.03 (5)	0.80 ± 0.05 (3)	0.99 ± 0.19 (3)	0.91 ± 0.08 (4)
GABRA5 / α5β3γ2	α5V204I	1.04 ± 0.14 (8)	0.95 ± 0.06 (17)	1.13 ± 0.09 (13)	0.97 ± 0.10 (9)	0.84 ± 0.05 (3)	1.05 ± 0.06 (11)
	α5W280R	0.667± 0.06 (5)	0.83 ± 0.04 (10)	0.94± 0.07 (13)	0.80 ± 0.08 (5)	0.77 ± 0.10 (4)	0.88 ± 0.05 (11)
	α5S402A	0.81 ± 0.11 (3)	0.90 ± 0.10 (3)	1.03 ± 0.01 (3)	0.97 ± 0.16 (3)	1.11 ± 0.05 (3)	0.98 ± 0.02 (3)
	α5P453L	0.85 ± 0.07 (11)	0.91 ± 0.07 (11)	1.01 ± 0.10 (7)	0.92 ± 0.06 (10)	1.16 ± 0.22 (4)	0.94 ± 0.10 (9)
GABRA6 / α6β2γ2	α6Q237R	0.96 ± 0.05 (5)	1.09 ± 0.06 (6)	0.93 ± 0.04 (6)	1.00 ± 0.10 (6)	1.01 ± 0.08 (6)	1.00 ± 0.04 (5)
GABRB1 / α1β1γ2	β1H421Q	1.12 ± 0.12 (3)	1.58 ± 0.39 (4)	1.10 ± 0.07 (3)	1.00 ± 0.21 (3)	1.30 ± 0.18 (3)	0.84 ± 0.01 (3)
GABRB2 / α1β2γ2	β2R293W	0.83 ± 0.03 (3)	0.95 ± 0.10 (3)	1.30 ± 0.12 (3)	1.04 ± 0.07 (3)	1.03 ± 0.15 (3)	1.10 ± 0.02 (3)
	β2R354C	0.95 ± 0.10 (4)	1.03 ± 0.09 (4)	1.27 ± 0.10 (4)	1.22 ± 0.09 (4)	1.06 ± 0.01 (3)	0.86 ± 0.02 (3)

GABRG1 / α1β2γ1	γ1S16R	1.03 ± 0.24 (4)	0.89 ± 0.06 (5)	1.06 ± 0.03 (5)	0.71 ± 0.07 (3)	1.02 ± 0.13 (5)	0.91 ± 0.09 (5)
	γ1S414N	1.04 ± 0.16 (5)	1.28 ± 0.17 (6)	1.22 ± 0.17 (6)	0.85 ± 0.06 (5)	1.01 ± 0.14 (4)	0.98 ± 0.30 (3)
GABRG3 / α5β3γ3	γ3A303T	1.22 ± 0.18 (3)	1.02 ± 0.14 (4)	0.74 ± 0.14 (5)	0.81 ± 0.02 (3)	1.34 ± 0.27 (4)	1.30 ± 0.28 (4)

Values are expressed as mean ± S.E.M. *n* = parenthesis. No significant differences from wild type (wt) after analysis of number of comparisons per gene by using one-way ANOVA with Dunnett's multiple comparisons test and unpaired t test (two-tailed) for single comparisons ($p > 0.05$). GEC = genetic epilepsy case.
