# Supplementary material for: Deleterious Rare Variants Reveal Risk for Loss of GABAA Receptor Function in Patients with Genetic Epilepsy and in the General Population
Source: PLoS One. 2016 Sep 13;11(9):e0162883. doi: 10.1371/journal.pone.0162883 (PMC5021343; doi:10.1371/journal.pone.0162883)
Supplement: S2 Table — (PDF) [file pone.0162883.s004.pdf]

**S2 Table**

**No Effects of ESP variants on the expression properties of α1β3γ2 $GABA_A$ receptors**

| | | Surface | | | Total | | |
|---|---|---|---|---|---|---|---|
| Gene | Variant | α | β | γ | α | β | γ |
| *GABRA1* | α1D9E | 1.09 ± 0.11 (3) | 0.98 ± 0.09 (3) | 1.12 ± 0.03 (3) | 0.82 ± 0.07 (3) | 0.74 ± 0.11 (3) | 1.14 ± 0.09 (3) |
| | α1P29S | 0.64 ± 0.10 (3) | 0.87 ± 0.08 (3) | 0.99 ± 0.25 (3) | 0.36 ± 0.02 (3) | 0.51 ± 0.12 (3) | 0.91 ± 0.33 (3) |
| | α1H129Y | 0.90 ± 0.31 (3) | 0.90 ± 0.18 (3) | 1.15 ± 0.23 (3) | 0.89 ± 0.03 (3) | 0.64 ± 0.15 (3) | 0.82 ± 0.20 (3) |
| | α1R147W | 1.08 ± 0.20 (3) | 1.05 ± 0.15 (3) | 1.26 ± 0.27 (3) | 0.85 ± 0.07 (3) | 0.65 ± 0.15 (3) | 0.89 ± 0.29 (3) |
| | α1T371I | 1.0 ± 0.17 (3) | 0.91 ± 0.14 (3) | 0.97 ± 0.12 (3) | 0.76 ± 0.05 (3) | 0.54 ± 0.08 (3) | 0.80 ± 0.14 (3) |
| | α1D383N | 1.20 ± 0.22 (3) | 0.76 ± 0.04 (3) | 1.21 ± 0.15 (3) | 1.04 ± 0.11 (3) | 0.73 ± 0.05 (3) | 1.16 ± 0.37 (3) |
| | α1P409S | 1.05 ± 0.18 (3) | 1.34 ± 0.42 (3) | 1.11 ± 0.17 (3) | 1.16 ± 0.31 (3) | 1.11 ± 0.46 (3) | 0.95 ± 0.32 (3) |
| | α1K410R | 1.10 ± 0.19 (3) | 0.96 ± 0.13 (3) | 1.18 ± 0.19 (3) | 1.14 ± 0.23 (3) | 0.69 ± 0.10 (3) | 0.88 ± 0.21 (3) |
| | α1T441M | 1.08 ± 0.17 (3) | 0.88 ± 0.18 (3) | 1.15 ± 0.39 (3) | 1.24± 0.55 (3) | 0.91 ± 0.20 (3) | 0.96 ± 0.30 (3) |
| *GABRB3* | β3R194Q | 0.96 ± 0.05 (4) | 0.77 ± 0.09 (4) | 1.04 ± 0.14 (4) | 0.94 ± 0.12 (4) | 0.40 ± 0.12 (3) | 0.71 ± 0.23 (4) |
| | β3D197N | 1.04 ± 0.52 (3) | 1.06 ± 0.21 (3) | 1.15 ± 0.11 (3) | 0.78 ± 0.03 (3) | 0.42 ± 0.22 (3) | 0.61 ± 0.19 (3) |

| | | | | | | | |
|---|---|---|---|---|---|---|---|
| | β3V200I | 0.88 ± 0.09 (4) | 0.93 ± 0.15 (3) | 1.07 ± 0.20 (3) | 0.89 ± 0.14 (3) | 0.81 ± 0.35 (3) | 1.14 ± 0.49 (4) |
| | β3R221K | 1.54 ± 0.48 (4) | 0.97 ± 0.11 (3) | 0.78 ± 0.16 (3) | 0.76 ± 0.07 (3) | 0.74 ± 0.28 (3) | 1.11 ± 0.50(4) |
| | β3R238W | 1.13 ± 0.21 (4) | 0.65 ± 0.33 (5) | 0.85 ± 0.23 (4) | 1.06 ± 0.35 (4) | 0.41 ± 0.32 (3) | 1.07 ± 0.69 (4) |
| | β3D387N | 0.86 ± 0.05 (4) | 1.09 ± 0.24 (4) | 1.10 ± 0.07 (4) | 0.78 ± 0.16 (4) | 1.56 ± 1.15 (3) | 1.22 ± 0.34(4) |
| | β3I448V | 1.11 ± 0.11 (4) | 1.54 ± 0.16 (4) | 0.99 ± 0.17 (4) | 0.71 ± 0.07 (4) | 2.29 ± 1.13 (3) | 1.36 ± 0.52 (4) |
| *GABRG2* | γ2L57F | 1.29 ± 0.04 (4) | 1.52 ± 0.29 (4) | 1.04 ± 0.13 (4) | 1.06 ± 0.08 (4) | 0.92 ± 0.29 (3) | 1.47 ± 0.46 (3) |
| | γ2A402T | 1.11 ± 0.13 (4) | 1.40 ± 0.30 (4) | 1.13 ± 0.15 (4) | 1.10 ± 0.07 (4) | 1.47 ± 0.44 (3) | 1.76 ± 0.36 (3) |

Values are expressed as mean ± S.E.M of $n = 3$-5, with differences among means not statistically significant ($p > 0.05$, one-way ANOVA with Dunnett's multiple comparisons test). ESP = Exome Sequencing Project.
