# Supplementary material for: Deleterious Rare Variants Reveal Risk for Loss of GABAA Receptor Function in Patients with Genetic Epilepsy and in the General Population
Source: PLoS One. 2016 Sep 13;11(9):e0162883. doi: 10.1371/journal.pone.0162883 (PMC5021343; doi:10.1371/journal.pone.0162883)
Supplement: S3 Table — (PDF) [file pone.0162883.s005.pdf]

S3 Table

Distribution of missense *GABR* variants by deleteriousness and effect on GABA-evoked currents		
	Damaging	Benign or possibly damaging
Reduced current	W280R<br>R293W<br>A303T<br>R354C<br>H129Y<br>T441M<br>V200I<br>I448V<br>L57F	H372P<br>V204I<br>Q237R<br>P29S<br>P409S<br>R194Q<br>R221K<br>R238W<br>D387N
No effect	R147W	T20I<br>P453L<br>A19T<br>S402A<br>H421Q<br>S16R<br>S414N<br>D9E<br>T371I<br>D383N<br>K410R<br>D197N<br>A402T

Data analyzed	damaging	benign	Total
I effect	9	9	18
no-effect	1	13	14
Total	10	22	32
Fisher's exact test			
P value	0.0189		
P value summary	*		
One- or two-tailed	Two-tailed		
Statistically significant? (alpha<0.05)	Yes		
