# Supplementary material for: Deleterious Rare Variants Reveal Risk for Loss of GABAA Receptor Function in Patients with Genetic Epilepsy and in the General Population
Source: PLoS One. 2016 Sep 13;11(9):e0162883. doi: 10.1371/journal.pone.0162883 (PMC5021343; doi:10.1371/journal.pone.0162883)
Supplement: S4 Table — (PDF) [file pone.0162883.s006.pdf]

S4 Table

Distribution of missense *GABR* variants by GABA$_A$ receptor structural domains and GABA-evoked currents			
	NT/TM	SP/CL	
Reduced current	W280R<br>R293W<br>A303T<br>H129Y<br>T441M<br>V200I<br>I448V<br>L57F<br>V204I<br>Q237R<br>P29S<br>R194Q<br>R221K<br>R238W	R354C<br>H372P<br>P409S<br>D387N	
No effect	R147W<br>P453L<br>D197N	T20I<br>A19T<br>S402A<br>H421Q<br>S16R<br>S414N<br>D9E<br>T371I<br>D383N<br>K410R<br>A402T	
Data analyzed	NT-TM	SP-CL	Total
I effect	14	4	18
no-effect	3	11	14
Total	17	15	32
Fisher's exact test			
P value	0.0036		
P value summary	**		
One- or two-tailed	Two-tailed		
Statistically significant? (alpha<0.05)	Yes		

NT = N-terminal. TM = transmembrane. SP = signal peptide. CL = M3/M4 cytoplasmic loop.
